# Life Cycle Assessment (LCA) Challenges in Evaluating Emerging Battery Technologies: A Review

**DOI:** 10.3390/ma18184321

**Published:** 2025-09-15

**Authors:** Renata Costa

**Affiliations:** Chemistry Research Centre of the University of Porto/Institute of Molecular Sciences (CIQUP-IMS), Faculty of Sciences, University of Porto, Rua do Campo Alegre s/n, 4169-007 Porto, Portugal; renata.costa@fc.up.pt

**Keywords:** post-lithium batteries, life cycle assessment (LCA), circular economy, next-generation batteries, battery sustainability assessment

## Abstract

As the demand for more efficient energy storage solutions grows, emerging battery chemistries are being developed to complement or potentially replace conventional lithium-ion technologies. This review explores the circular economy potential of sodium (Na), magnesium (Mg), zinc (Zn), and aluminum (Al) battery systems as alternative post-lithium configurations. Through a comparative literature analysis, it identifies key barriers related to material complexity, recovery efficiency, and regulatory gaps, while highlighting opportunities for design improvements and policy alignment to enhance sustainability across battery life cycles. However, end-of-life (EoL) material recovery remains constrained by complex chemistries, low technology readiness levels, and fragmented regulatory frameworks. Embedding materials/battery design principles, transparent life cycle assessment (LCA) data (e.g., publishing LCAs in open repositories using a standard functional unit), and harmonized policy early could close material loops and transform the rising post-lithium battery stream into a circular-economy resource rather than a waste burden.

## 1. Introduction

**Figure 1:**
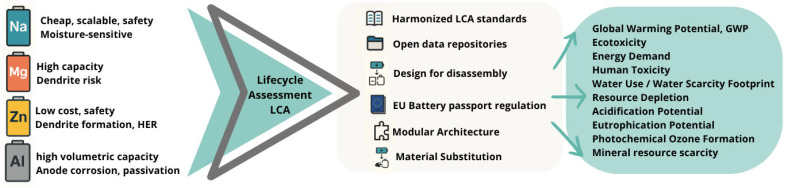
Schematic overview of emerging battery chemistries (Na, Mg, Zn, Al) and their key attributes, integrated into a life cycle assessment (LCA) framework.

The review spans the life cycle of four emerging battery chemistries, including sodium-, magnesium-, zinc-, and aluminum-battery technologies, mapping how their distinct chemistries affect second-life prospects and overall circular-economy value. It contrasts their electrochemical architecture against electrode/electrolyte materials, highlighting Na moisture-sensitive electrolytes, Mg dendrite risk, Zn aqueous separation hurdles, and Al corrosion issues. A dedicated section to each technology synthesizes recent life cycle assessment studies (LCA), tracing how inconsistent functional units (FU), inventory gaps, and evolving EU “battery passport” rules skew comparability and policy relevance. Finally, the analysis layers in economics and governance, quantifying how fluctuating critical-raw-material prices, geopolitical burdens, extended-producer-responsibility, and design-for-disassembly standards could accelerate closed-loop supply chains. The strategic levers discussed include modular cell formats to simplify dismantling, price signals for secondary metals, and harmonized eco-design metrics that reward recyclability alongside performance (Figure 1).

The review calls for harmonized LCA practices with standardized system boundaries, FU, and data-quality criteria to enable clearer, chemistry-agnostic comparisons and policy insights.

## 2. Methodology

The focus of the review will be on recent publications, mostly published within the last five years, reporting the technological challenges, environmental performance, and circular economy potential of emerging post-lithium battery chemistries, especially through the lens of LCA studies. Peer-reviewed articles, industrial reports, and regulatory documents were selected from major academic databases (e.g., Scopus, Web of Science, and ScienceDirect), focusing on Na-, Mg-, Zn-, and Al-battery technologies, while Li batteries were used as a proper benchmark. The selection criteria prioritized sources that addressed LCA, material criticality, recyclability, and end-of-life (EoL) management. The analysis framework categorized findings under key themes, including technical challenges, environmental impact, economic feasibility, and regulatory landscape. Emphasis was placed on the material composition, design complexity, recovery efficiency, and alignment with circular economy principles. The work does not intend to be exhaustive; rather, it aims to highlight and identify critical methodological and sustainability challenges associated with the LCA of emerging battery technologies.

To ensure clarity and consistency in interpreting LCA results, it is essential to explicitly define the environmental indicators commonly used to evaluate battery technologies. ISO 14040:2006 [1] (confirmed in 2022) defines the framework for LCA, covering goal and scope, inventory, impact assessment, and interpretation. It is a widely used tool to evaluate the environmental impacts of products from cradle to grave. Studies commonly employed software such as SimaPro 8.3-10.1 and OpenLCA 2.0-2.5, alongside various impact assessment methods and tools, to evaluate the environmental impacts of battery production and use. The ReCiPe method is one of the most widely used LCA methodologies, and it translates LCA inventory data such as emissions, resource use, and energy consumption into environmental impact scores. ReCiPe works at two main levels: (a) midpoint indicators, which represent specific environmental problems such as Global Warming Potential, Acidification Potential, or Eutrophication Potential, and (b) endpoint indicators, which aggregate midpoint impacts into damage-oriented categories affecting human health, ecosystem quality, and resource availability. The endpoint indicators measure the ultimate damage caused by environmental stressors at the end of the cause-and-effect chain. While they provide a clear picture of impacts, they come with greater uncertainty [2].

Table 1 summarizes the main LCA indicators, environmental dimensions, and relevance to battery studies based on the Recipe method.

## 3. Emerging Post-Lithium Chemistries

The growing urgency to diversify beyond lithium-ion batteries has led to intensified research into a range of alternative chemistries that promise to be more sustainable, cost-effective, and safer. Among the most actively studied battery configurations are Na-ion [3,4,5,6,7,8,9,10], Mg-ion [11,12,13,14,15,16,17,18], Zn-ion [19,20,21,22,23,24,25,26], and Al-ion technologies [27,28,29,30,31,32,33]. Na-ion batteries have seen commercial momentum, with major manufacturers such as CATL (Ningde, China) launching mass-produced Na-ion cells by late 2025. Concurrently, Mg-ion systems are gaining traction due to their theoretical volumetric capacities and ion safety profile, achieving promising performance metrics in lab settings. These systems generally rely on more earth-abundant materials, offering the potential to reduce dependence on geographically concentrated and often ethically problematic supply chains. In addition to material availability, some of these chemistries, particularly ionic liquid-based electrolytes, offer intrinsic safety advantages due to their non-flammability, making them appealing for stationary storage and other risk-sensitive applications. Emerging beyond these systems, metal–air and metal–sulfur batteries have also gained considerable attention for their exceptional theoretical energy densities and the use of earth-abundant, low-cost materials. Metal–air batteries, particularly exploit oxygen from the atmosphere as the cathodic reactant, thereby minimizing the mass of active materials required and offering gravimetric energy densities approaching those of fossil fuels [34]. However, challenges such as oxygen crossover, sluggish oxygen reduction and evolution reactions (ORR/OER), and electrolyte instability have limited their practical deployment to date. Similarly, metal–sulfur batteries, with Li-S and Na-S chemistries at the forefront, present a promising alternative due to sulfur’s high natural abundance, low cost, and intrinsic safety advantages [35]. Their theoretical energy densities surpass those of conventional Li-ion batteries, making them attractive for long-duration energy storage and heavy-duty applications. Yet, persistent issues like polysulfide shuttle effects, volume expansion, and cycle life degradation remain significant barriers to commercialization.

Each of these emerging battery technologies presents a unique electrochemical mechanism and materials profile, leading to different performance characteristics and lifecycle behaviors. For instance, Na-ion batteries closely mirror the architecture of Li-ion systems, which may ease their integration into existing production lines, while multivalent systems like Mg-ion and Al-ion batteries promise higher volumetric capacities but are hindered by slow ion transport and compatibility issues [15,36]. Understanding the fundamental design and operational principles of these chemistries is essential for assessing the specific challenges and opportunities they pose in terms of recyclability and circular economic integration [37]. Recent research by Picatoste et al. [38] emphasizes that while circular design principles are beginning to shape Li-ion battery development, the establishment of tailored circularity criteria and product-level indicators for other chemistries is significantly lagging. This is particularly problematic because stage design decisions greatly influence recyclability, second-life potential, and environmental impact of batteries across their life cycle chain value.

### 3.1. Sodium-Battery Systems

#### 3.1.1. Materials and Mechanisms

**Figure 2:**
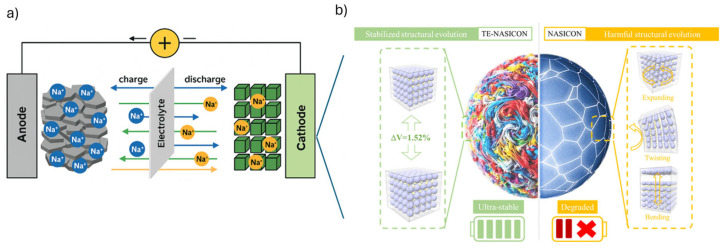
**a**) Scheme represents the working principle of sodium-ion (Na-ion) batteries, **b**) multi-Enhanced NASICON cathode (Na_4_Cr_0.7_Mn_0.65_Fe_0.1_Ni_0.1_V_0.2_Al_0.2_(PO_4_)_3_, ME-NASICON), achieving high voltage, high entropy, and exceptional cycling stability (reproduced from reference [40]. Copyright 2025, American Chemical Society).

Na-ion batteries are gaining widespread attention as a sustainable and economically viable alternative to Li-ion batteries, stemming from the abundant availability of sodium and its lower environmental extraction footprint compared to lithium [5]. As the demand for energy storage systems grows, driven by the expansion of renewable energy and the need for grid flexibility, Na-ion batteries offer a cost-effective solution with reduced dependence on geographically constrained critical materials [39]. The working principle of Na-ion batteries mirrors that of Li-ion, involving the reversible movement of sodium ions (Na^+^) between the anode and cathode through an electrolyte during charge and discharge cycles. When charging, Na^+^ ions are deintercalated from the cathode and intercalated into the anode, and the process is reversed during discharge, releasing stored electrical energy (Figure 2) [6].

However, sodium’s larger ionic radius and higher atomic mass introduce specific challenges, such as slower diffusion and greater mechanical strain on host materials [41,42]. These factors require the development of specially engineered electrode materials that can endure repeated cycling while maintaining structural integrity and capacity. Banerjee et al. [43] significantly advance this drawback by providing a comprehensive assessment of Na_3_V_2_(PO_4_)_3_ (NVP)-based cathode materials. The work highlights how compositing NVP with conductive polymers and nanostructured materials can overcome its inherent limitations, such as low electronic conductivity and volume expansion, leading to enhanced cycling stability, rate capability, and energy density. The work also outlines optimized synthesis routes and advanced surface engineering techniques, positioning NVP composites as highly promising candidates for scalable, high-performance storage applications. Hard carbon (HC) has significantly advanced the performance and viability of Na-ion batteries by addressing the fundamental challenges posed by sodium’s larger ionic radius and sluggish kinetics compared to lithium [3]. On the anode side, HC is a leading material due to its disordered structure and expanded interlayer spacing, which accommodate Na^+^ ions effectively. Accordingly, it provides stable performance, with specific capacities around 300 mAh g^−1^ and a cycle life up to 3000 cycles [44]. Carbon amorphous structure, rich in defects and nanopores, provides abundant active sites and expanded interlayer spacing, which enhances sodium ion storage capacity, facilitates ion diffusion, and improves rate performance [45]. Unlike graphite, HC is capable of accommodating sodium through multiple mechanisms, including intercalation, surface adsorption, and pore filling. Importantly, the sodium-storage mechanism in HC is not adequately described by a single model. Instead, multiple conceptual frameworks have emerged to capture the complexity of Na^+^ interaction with HC structures, which include the “Insertion-Adsorption” model, the “Adsorption-Intercalation” model, the “Three-Stage” model, and the “Adsorption-Filling” model, each one emphasizing a different combination of surface adsorption, interlayer intercalation, and nanopore filling.

Researchers are also exploring other anode candidates like phosphorus-based materials, offering extremely high theoretical capacities, as well as metal oxides and transition metal dichalcogenides such as MoS_2_ [46,47,48]. Each of these alternatives comes with its trade-offs in terms of capacity, cycling stability, and manufacturability. For example, Yang et al. [48] introduced a novel 3D MoS_2_/graphene oxide composite anode with a hierarchical porous structure, significantly enhancing sodium-ion diffusion, electrical conductivity, and mechanical stability, thus achieving a high reversible specific capacity of 525 mAh g^−1^ with long-term cycling stability over 3000 cycles. In contrast, the MXene@MoS_2_ composite presents a lower specific capacity of 257.8 mA h g^−1^ after 1000 cycles at a current density of 1 A g^−1^ with a capacity retention of 95.7% [49].

**Figure 3:**
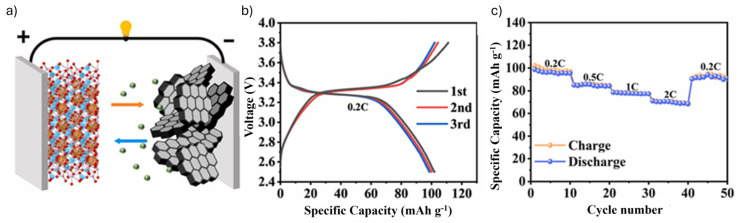
**a**) Diagrammatic drawing, **b**) charge–discharge curves for the first three cycles at 0.2 C, and **c**) rate performance of the NVP@FG||HC full cell. Adapted from reference [51] with permission from Elsevier.

The cathode plays a critical role in determining the overall energy density and voltage of Na-ion batteries. Zhu et al. [40] developed a multielement-doped high-entropy NASICON cathode for Na-ion batteries, achieving high voltage, exceptional cycling stability, and a specific capacity of 166.98 mAh g^−1^ with minimal structural degradation, enabling long-lifetime, high-energy-density energy storage (Figure 2b). Layered transition metal oxides, such as NaCrO_2_, NaFeO_2_, NaCuO_2_, NaNiO_2_, NaCoO_2_, NaVO_2_, NaMnO_2_, have shown promising performance due to their high specific capacities [50]. However, it is reported to be a challenge related to structural degradation over time. Polyanionic compounds like Na_3_V_2_(PO_4_)_3_ offer high thermal stability and well-defined voltage plateaus, making them suitable for applications that require safety and longevity. Liu et al. [51] developed a hierarchical fragmented Na_3_V_2_(PO_4_)_3_@reduced graphene oxide (NVP@FG) composite that significantly enhances Na^+^ diffusion, electron conductivity, and structural stability, achieving high reversible specific capacity (114.5 mAh g^−1^), excellent rate performance, and remarkable cycling durability with 97.5% capacity retention after 1800 cycles (Figure 3).

Prussian blue analogs (PBAs) are another compelling class of cathode materials, particularly due to their low cost, ease of synthesis, and structural openness, which facilitates fast sodium-ion diffusion [52]. Despite their potential, PBAs still face challenges related to moisture sensitivity and vacancy-related capacity losses. To mitigate that, Li et al. [53] developed a universal fast-sintering strategy that directly converts PBAs into high-performance layered oxide cathodes for sodium-ion batteries, significantly reducing energy consumption, enabling effective reuse of unqualified PBAs, and delivering competitive electrochemical performance with excellent structural reversibility and scalability.

Na-ion battery systems predominantly use liquid electrolytes, typically based on sodium salts such as NaPF_6_ (sodium hexafluorophosphate) dissolved in carbonate solvents [54]. Innovations in solid-state and ionic liquid electrolytes are being pursued to improve thermal stability and mitigate risks associated with flammability and electrolyte degradation. As an example, the comprehensive analysis and strategic framework performed by Lee et al. [55] for developing high-performance solid-state sodium batteries through the design and optimization of inorganic solid electrolytes (ISEs), focusing on enhancing ionic conductivity, electrochemical stability, and interfacial compatibility. By examining key factors such as ion migration mechanisms, defect chemistry, and electrode/ISE interface engineering, the authors identified critical pathways to overcome limitations like narrow electrochemical stability windows and Na dendrite formation. Recent breakthroughs are reported in electrolyte materials, including β-alumina, NASICON, sulfide, and halide-based electrolytes, and novel synthesis methods and structural modifications that significantly improve performance, offering a clear and promising pathway for the practical commercialization of safe, efficient, and scalable solid-state electrolytes [56,57,58,59].

In terms of applications, Na-ion batteries are especially attractive for large-scale stationary energy storage, where cost, safety, and lifespan are more critical than compactness or high energy density [60]. Na-ion batteries’ competitive cost per kilowatt-hour makes them ideal for grid-scale installations, renewable energy integration, and backup power systems. While their energy density is generally lower than that of Li-ion battery technology, Na-ion batteries offer adequate performance for emerging markets such as urban electric mobility (e.g., scooters and low-speed electric vehicles), and in specific portable electronic devices where size is less of a constraint [61,62]. Yao et al. [63] analyzed the future cost competitiveness of Na-ion batteries compared to Li-ion using a detailed modeling approach that incorporates material learning curves and engineering roadmaps. The authors concluded that Na-ion batteries could reach price parity or advantage over low-cost Li-ion variants by the 2030s, but only if energy density improves and supply chain conditions shift, e.g., lithium or graphite price increases. The study finds that technical advancements, rather than scale alone, are key to lowering Na-ion costs, highlighting the importance of research on cathode design, anode alternatives, and voltage optimization.

#### 3.1.2. LCA Na-Batteries

Na-ion batteries have reached a reasonable level of technological maturity that allows for more detailed LCA modeling, including full cradle-to-gate and cradle-to-grave assessments. The breadth of recent studies on Na-ion systems spanning different chemistries, energy sources, and production methods demonstrates a strong momentum in both academic and industrial settings toward understanding and improving their environmental performance. Table 2 aims to provide a structured and systematic overview of LCA studies conducted on Na-ion batteries, organized according to the four standard phases of the LCA framework: goal and scope definition, LCA inventory analysis, impact assessment, and interpretation.

**Figure 4:**
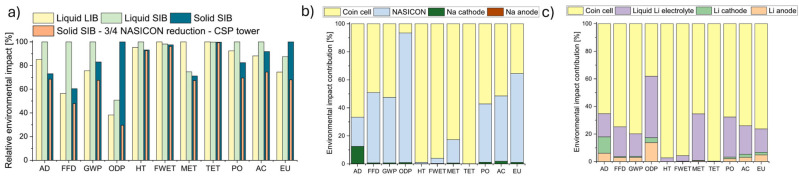
**a**) LCA results of liquid Li, liquid Na, and solid Na batteries in relative environmental impact. Solid Na battery includes a sensitivity analysis with the energy provided by tower CSP technology, **b**) environmental impact contributions of solid, and **c**) liquid Na-batteries. Adapted from reference [65] with permission from Elsevier.

Wickerts et al. [64] present a forward-looking cradle-to-gate LCA of two sodium-ion battery cells designed with abundant materials. The goal was to evaluate their environmental and resource impacts at a large production scale, especially in comparison with conventional Li-ion batteries, which often rely on critical raw materials such as cobalt and lithium. The two modeled batteries utilize Prussian white cathodes and hard carbon anodes, with the functional unit set at 1 kWh of theoretical electricity storage and a specific energy density of 160 Wh kg^−1^. Data for cathode materials were obtained from a planned industrial-scale facility, while other inventory data were built using a standardized prospective 8-step procedure, enabling more accurate estimation of future Na-batteries supply chains. The key findings indicate that both Na-ion configurations show substantially lower impacts related to mineral resource scarcity compared to nickel-manganese-cobalt (NMC)-type Li batteries. Their GWP is comparable to that of Li-ion batteries, but highly sensitive to the electricity mix used during manufacturing. Importantly, sourcing fossil-free electricity and utilizing lignin-based hard carbon anodes significantly reduces the environmental impact. However, no electrolyte among those assessed emerged as clearly preferable, highlighting a need for further research into low-impact electrolytes suitable for Na-ion batteries. Batuecas et al. [65] present an LCA comparing solid-state Na-ion batteries, liquid Na-ion batteries, and conventional liquid Li-ion batteries at the lab scale. It evaluates their environmental impacts across multiple categories, including GWP, abiotic depletion, toxicity, and photochemical oxidation. The findings show that solid-state Na-ion batteries have lower impacts in toxicity and resource depletion categories, particularly when renewable energy sources like concentrated solar power (CSP) are used in production. However, they perform worse than Li-ion batteries in categories like ozone depletion and eutrophication. The study highlights the potential of solid Na-ion batteries as a sustainable alternative, especially when paired with green energy inputs and optimized material usage, though improvements in energy efficiency and electrolyte design are still needed (Figure 4a–c).

Zhang et al. [66] present a pLCA evaluating the future climate impacts of Na-ion batteries compared to Li-ion batteries under multiple development scenarios between 2020 and 2050. The analysis models considered three different Na-ion chemistries and two Li-ion chemistries, incorporating projections of future changes in battery performance and the electricity grid based on integrated assessment models. The results show that under optimal performance scenarios and cleaner energy conditions, Na-ion batteries can achieve GHG emissions that are equal to or lower than those of Li-ion batteries. The study emphasizes that decarbonizing the grid significantly reduces manufacturing impacts, shifting the environmental burden toward raw material production. It concludes that Na-ion batteries hold promising potential as environmentally competitive alternatives, especially as technology matures and cleaner production inputs become more widespread. Guo et al. [67] conduct a comparative LCA of Na-ion and lithium iron phosphate (LFP) batteries within the context of promoting carbon neutrality in electric vehicles (EVs). It evaluates the environmental impacts across the full battery life cycle, including production, use, and end-of-life, under four scenarios combining battery reuse (gradient utilization) and different recycling strategies. The findings show that while LFP batteries currently perform better environmentally during production, Na-battery systems offer advantages in long-term sustainability, especially with continued technological improvements. Among recycling methods, hydrometallurgical processes perform best, and gradient utilization effectively extends battery life and reduces environmental impact. The study concludes that Na-batteries, particularly when paired with efficient reuse and recycling systems, hold strong potential for sustainable EV battery development. Mozaffarpour et al. [68] present a cradle-to-gate LCA of Na-ion battery cathodes based on Na_3_MnCO_3_PO_4_ (NMCP), focusing on three different synthesis routes: hydrothermal, stirring-hydrothermal, and ball milling. The analysis evaluated key environmental indicators such as GWP, energy use, water consumption, and acidification. The results show that the ball milling method has the lowest overall environmental impact, primarily due to its shorter reaction time, lower energy consumption, and reduced material use. In contrast, the hydrothermal route exhibits the highest GWP and resource use. The study concludes that synthesis method selection is critical to minimizing the environmental footprint of Na-ion battery materials and identifies ball milling as the most sustainable route among those considered.

### 3.2. Magnesium-Battery Systems

#### 3.2.1. Materials, Progress, and Challenges

**Figure 5:**
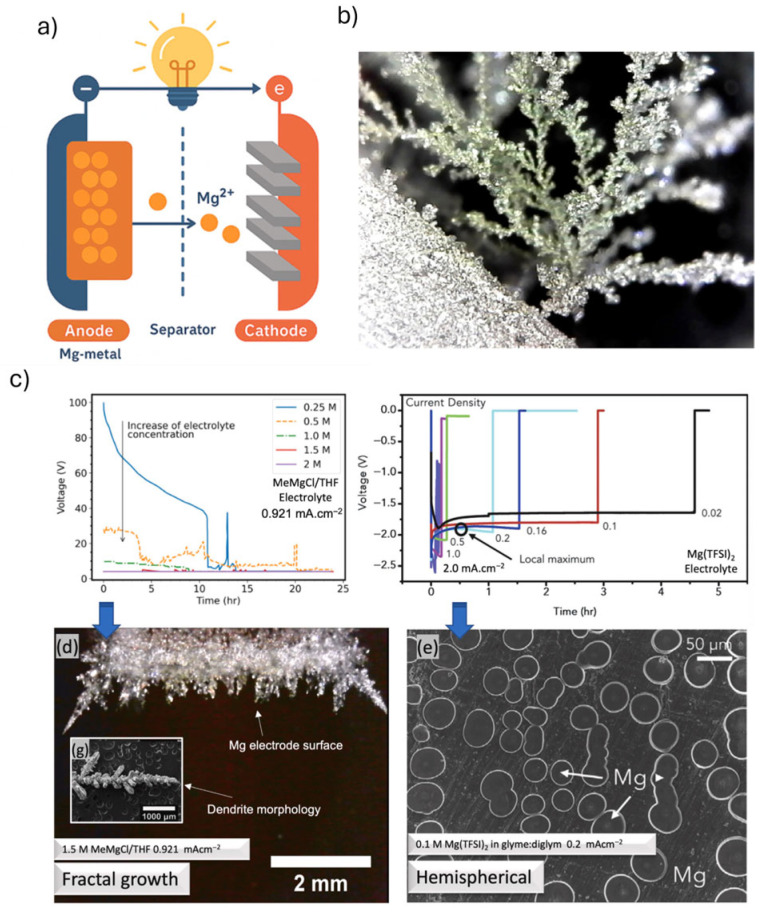
**a**) Schematic illustration of the working principle of a Mg-ion battery, **b**) dendritic magnesium deposits with fractal morphologies upon the galvanostatic electrodeposition of metallic Mg from Grignard reagents in symmetric Mg-Mg cells (reproduced from reference [81], copyright © 2018 American Chemical Society), **c**) galvanostatic curves for the electrodeposition of Mg in symmetric Mg-Mg cells under constant current conditions with MeMgCl, and Mg [TFSI] (organic-rich interface), **d**,**e**) represent the microstructures of the electrodes in each cell configuration after the galvanostatic transients (reproduced from reference [79], with Elsevier permission).

Magnesium, the lightest structural metal, is economically important due to its low density, high strength-to-weight ratio, and versatile properties, including excellent stiffness, damping, biocompatibility, hydrogen storage capacity, and high theoretical battery capacity [69]. Mg-batteries offer an impressive volumetric capacity of approximately 3833 mAh cm^−3^, nearly double that of Li-ion batteries, making them a highly attractive candidate for compact, high-energy-density storage applications [70]. Mg-ion technology operates by the reversible intercalation and deintercalation of divalent Mg^2+^ between the anode and cathode through an electrolyte [71]. Unlike Li-ion systems that transport monovalent Li^+^ ions, Mg-ion systems leverage magnesium’s divalency to potentially double the charge per ion, offering a higher theoretical volumetric energy density [72]. During discharge, Mg^2+^ ions migrate from the anode to the cathode, releasing electrons that flow through the external circuit, while upon charging, the process reverses (Figure 5) [15].

The Mg-ion batteries have long been considered a promising alternative to Li-ion systems, largely due to the belief that magnesium metal anodes were inherently resistant to dendrite formation [15,73]. This assumption stems from magnesium’s high surface diffusion rates and its stable hexagonal close-packed crystal structure, which favors smooth deposition [74]. For many years, this dendrite-free characteristic, combined with magnesium’s abundance and safety, made it a highly attractive candidate for next-generation rechargeable batteries [75,76]. However, since 2017, this narrative has shifted since researchers reported dendritic deposition of magnesium under specific conditions, revealing that while Mg may be less prone to dendrite formation than Li or Na, it is still not immune [77]. Mg dendrites, when they form, tend to be morphologically more complex and compact due to higher bonding energies and anisotropic growth behavior [78]. These findings have opened a new chapter in magnesium battery research, focused on understanding the conditions that lead to dendrite growth and developing strategies to prevent it (Figure 5b–e).

Dendrite formation in Mg-ion batteries begins during the electrodeposition process, where Mg^2+^ is reduced and deposited onto the anode surface [79]. Attari et al. describe that Mg dendrite formation arises primarily from high local overpotentials and transport limitations in the electrolyte, which lead to diffusion-limited aggregation and the growth of branched or hemispherical metal structures [79]. Incompatible electrolytes exacerbate the issue, increasing impedance and encouraging uneven deposition. Even in supposedly compatible systems, the adsorption of inactive species on the magnesium surface can impede ion transport and lead to localized current hotspots, further driving dendrite growth [80].

Anode key materials range from pure magnesium and alloyed forms (e.g., Mg-Al, Mg-Sn), which are employed for their conductivity and abundance, since alloying enhances ion transport and reduces polarization resistance, improving efficiency [82,83]. Promising anodes for Mg-ion batteries are alloy-based materials, with tin (Sn) and bismuth (Bi) standing out. Sn offers high capacity (~900 mAh g^−1^) but suffers from volume expansion and poor cycling; nanostructuring improves its stability [84]. Bi shows good reversibility (~350–385 mAh g^−1^) and compatibility with electrolytes, especially in nanostructured or composite forms [85]. Bi-Sn alloys further enhance performance through synergistic effects, while Sb, In, and Pb are less favorable due to issues like irreversibility, toxicity, or limited availability [86].

Mg-ion battery cathodes face significant challenges due to the high charge density and small ionic radius of Mg^2+^, which result in slow diffusion, strong Coulombic interactions, and lattice distortions. Sulfur-based materials (including sulfur–graphene and polyanionic composites) show promise with energy densities of 800–950 mAh g^−1^ and acceptable retention (~92% after 500 cycles) [15]. Sulfide-based cathodes like the Chevrel phase (Mo_6_S_8_) offer favorable ion mobility and structural stability, albeit with low voltages (<2 V) [87]. Oxide cathodes, such as manganese oxides and vanadates, deliver higher voltages (2–3 V) but often suffer from poor capacity retention and structural degradation [88,89]. Among highlighted materials, Mo_6_S_8_ remains a benchmark for stability and compatibility, while MnO_2_ polymorphs offer high initial capacities but degrade rapidly due to structural collapse [90].

Electrolytes are a critical component in Mg-ion batteries, as they directly impact ionic conductivity, compatibility with the Mg anode, and overall cell stability [91,92]. One of the key challenges is the formation of a passivation layer on the Mg metal surface, especially when using conventional salts and solvents. This layer, often impermeable to Mg^2+^, severely limits reversibility and cycle life [93,94,95]. To overcome these issues, researchers have explored various classes of electrolytes, including organic-liquid, aqueous, ionic-liquid, and solid-state systems. Organic-liquid electrolytes, particularly those based on ether solvents such as dimethyl ether, tetrahydrofuran, and glymes, have shown the best balance of Mg compatibility and ionic conductivity, though issues such as volatility and decomposition persist [96,97,98]. Efforts have also focused on designing new solvents (e.g., methoxyethylamine) and solvation structures to suppress passivation and enhance Mg^2+^ transport [99].

Mg-ion batteries are currently in the early stages of development, with research and prototyping efforts focused on applications such as EVs, stationary energy storage, and grid integration [70,100,101]. In the near term, Mg-ion batteries are being explored as a safer and more cost-effective solution for EVs, with promising results showing cycle stability above 90% after 500 cycles and energy densities nearing 1000 mAh g^−1^. Their robust safety profile and long service life also position them well for renewable energy buffering and grid-level storage, where reliability and sustainability are essential. Looking further ahead, these batteries may find use in consumer electronics, provided that current challenges in miniaturization and energy density scaling can be addressed. Despite their considerable potential, Mg-ion batteries face several hurdles on the path to commercialization. These include sluggish ion diffusion kinetics, the persistent issue of electrode passivation, and an underdeveloped supply chain for key materials and components. Nevertheless, ongoing advancements, particularly in the design of cathode materials and the development of more compatible, high-performance electrolytes, are gradually closing these gaps. As raw material costs continue to fall and safety concerns become more prominent in battery design, Mg-ion technology is poised to become a competitive and sustainable alternative in the energy storage market.

#### 3.2.2. LCA Mg-Batteries

Although the primary goal of this review was not to analyze metal–sulfur chemistries specifically, it was observed that the majority of published LCA studies on magnesium-based systems are limited to Mg-S battery configurations. This creates a methodological constraint, as the evidence base does not yet extend to other Mg battery architectures. To maintain consistency with the comparative approach of this work, the discussion therefore includes Mg-S studies as a proxy for Mg systems more generally, while also acknowledging the need for future assessments that address alternative Mg chemistries. This strategy ensures that the review captures the current state of knowledge without overstating generalizability, while still providing a coherent comparison across emerging post-lithium technologies.

Magnesium-based batteries have been explored in three distinct configurations: Mg-ion, Mg-air (Section 3.5), and Mg-S. Mg-ion batteries rely on the reversible intercalation of Mg^2+^ ions into host cathode structures such as oxides, vanadates, or Chevrel phases, offering the potential for high volumetric energy density due to the divalency of magnesium. However, their progress is hindered by sluggish ion diffusion and strong coulombic interactions within solid-state hosts, which limit capacity and cycling stability. In contrast, rechargeable Mg-S batteries employ elemental sulfur as the cathode and magnesium metal as the anode, taking advantage of sulfur’s high theoretical capacity (1675 mAh g^−1^) and the abundance and low toxicity of both active materials [102,103]. While Mg-S configuration promises lower resource-related impacts, it faces significant challenges such as polysulfide dissolution, high electrolyte reactivity, and poor cycle life.

Among all the battery technologies evaluated, Mg-based systems stand out for having the fewest published LCA studies to date, centered on Mg-S technology. Pinto-Bautista et al. [104] present an LCA for Mg-S batteries, thus evaluating their environmental performance across different configurations and chemistries. The study compares various cathode materials (including Chevrel phases and organic materials) with commercial Li-ion batteries to assess Mg-based batteries’ sustainability potential. The analysis reveals that Mg-S batteries generally have a lower environmental impact in terms of mineral resource scarcity and human toxicity, primarily due to the abundance and low toxicity of magnesium. However, the overall performance of Mg-based batteries is still challenged by lower energy density and immature technology compared to Li-ion technology. The study highlights the importance of optimizing cell design and sourcing renewable energy to improve Mg-based sustainability and concludes that with further development, it could offer a promising, resource-resilient alternative to Li-ion batteries. Montenegro et al. [105] present the LCA of Mg-S batteries, focusing on their environmental performance compared to Li-ion batteries. The analysis evaluates multiple environmental impact categories, including GWP, human toxicity, and resource depletion, using a cradle-to-gate scope. The Mg-S battery is modeled at a lab-scale prototype level and features a magnesium anode and a sulfur cathode. The results show that Mg-S batteries could offer environmental advantages due to the abundance and low toxicity of magnesium and sulfur, particularly in reducing mineral resource scarcity and human toxicity impacts. However, their overall performance is currently limited by lower energy density and high impact from electrolyte synthesis. The study concludes that while promising, Mg-S batteries require further technological optimization and improved material processing to be environmentally competitive alternatives to Li-ion technology. While Montenegro et al. provide a technical-material baseline for Mg-S batteries at early development, Pinto-Bautista et al. deliver a systems-level projection of their performance in real-world electric mobility, showing greater confidence in their long-term environmental viability.

### 3.3. Zinc-Battery Systems

#### 3.3.1. Materials, Stability, and Practical Applications

**Figure 6:**
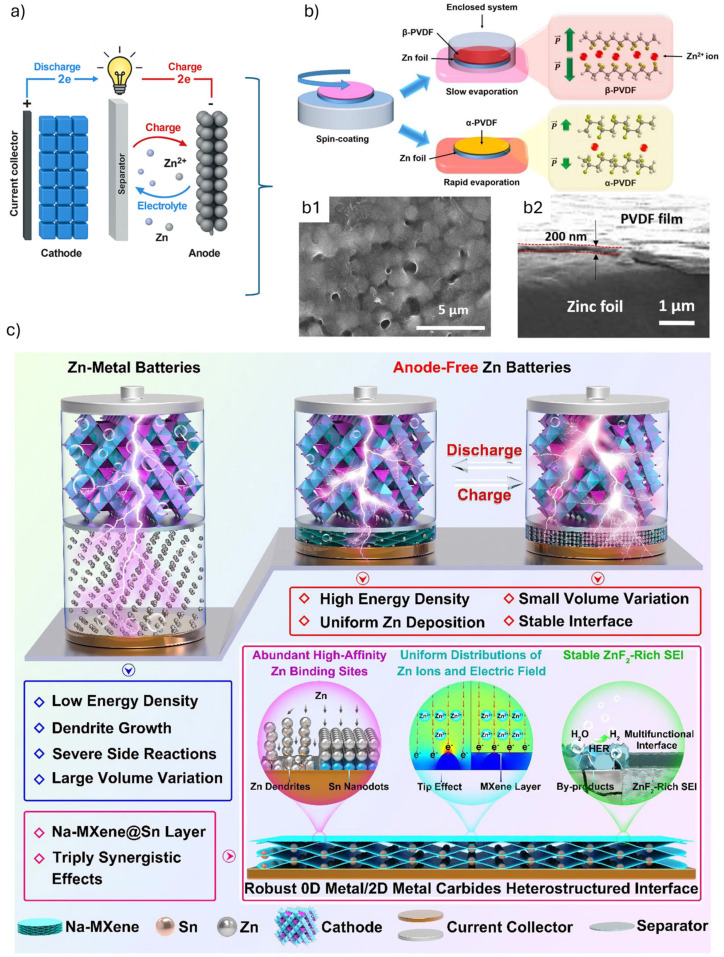
**a**) Schematic illustration of the working principle of a Zn-ion battery. Red and blue arrows indicate charging and discharging pathways, respectively, **b**) schematic illustration of the fabrication of β-phase poly (vinylidene difluoride, β-PVDF) and α-phase poly (vinylidene difluoride, α-PVDF), **b1**) SEM image of β-PVDF@Zn anode, **b2**) cross-sectional morphology of a β-PVDF@Zn anode (reproduced from reference [108], with Elsevier permission); and **c**) 0D metal/2D metal carbide heterointerface with triply synergistic effects is designed to regulate interfacial chemistry for aqueous anode-free Zn batteries (reproduced from reference [109], with Elsevier permission).

Zn-ion batteries operate based on the reversible plating and stripping of zinc ions (Zn^2+^) between the anode and cathode, typically within an aqueous electrolyte system. The working principle hinges on several key electrochemical and physical processes that collectively enable energy storage and release [106]. The anode in Zn-ion batteries is usually composed of metallic zinc, in which, during discharge, zinc undergoes oxidation, releasing Zn^2+^ ions into the electrolyte and generating electrons: $Zn\to {Zn}^{2+}+{2e}^{-}$ The electrons move through an external circuit to power devices, while Zn^2+^ ions migrate through the electrolyte towards the cathode. Accordingly, at the cathode, Zn^2+^ ions are inserted (intercalated) into the host material, typically transition metal oxides like MnO_2_ or vanadium-based compounds [107]. The cathode materials accept the Zn^2+^ ions and the electrons from the external circuit, undergoing reduction: ${MnO}_{2}+{Zn}^{2+}+2e^{-}\to {Zn}_{x}{MnO}_{2}$. Upon charging, this process is reversed, in which Zn^2+^ ions leave the cathode and are reduced back to metallic zinc at the anode (Figure 6).

The advancement of aqueous Zn-ion batteries presents persistent challenges associated with zinc anodes, such as dendrite growth, hydrogen evolution reaction (HER), corrosion, and passivation. Alloying strategies become an effective solution to these problems, highlighting how introducing heteroatoms into the zinc matrix can suppress dendrite formation, enhance interfacial kinetics, and improve mechanical and electrochemical stability [110]. Alloy design strategies include homogeneous bulk alloys, surface alloy engineering, functional alloying, heterogeneous composites, gradient alloying, and layered architectures, detailing their mechanisms and performance benefits. The interfacial modification of zinc metal anodes with alloys influences key aspects like nucleation kinetics, charge distribution, corrosion resistance, and hydrogen suppression by modifying the electronic structure and interfacial characteristics of zinc anodes [111]. Tao et al. [108] present an in-depth summary of various surface modification techniques developed to enhance the performance of Zn metal anodes in aqueous Zn-ion batteries. The surface modification serves as a practical and scalable strategy to mitigate issues related to dendrite growth, hydrogen evolution reactions, corrosion, and passivation. Solvent casting, including spin coating and doctor blading, stands out for its scalability and simplicity, enabling uniform coatings like PVDF (poly-1,1-difluoroethene) and cyanoacrylate layers that regulate Zn ion deposition and limit side reactions. Alternatively, wet chemistry, particularly electrodeposition and Metal–Organic Frameworks (MOF) integration, offers low-cost and uniform film formation with porous and hydrophilic surfaces that facilitate ion diffusion and suppress dendrites. Although aqueous electrolytes enhance the safety of Zn-ion batteries, these types of electrolytes are prone to HER, especially in alkaline conditions, leading to gas buildup, increased internal pressure, and potential battery failure. In neutral or mildly acidic electrolytes, HER is less favorable but still occurs due to Zn^2+^ hydrolysis and H^+^ reduction, which depletes the electrolyte, reduces conductivity, and impairs zinc plating/stripping efficiency. HER competes with zinc deposition in aqueous media, reducing Coulombic efficiency [112,113,114,115].

The corrosion phenomena observed in aqueous Zn-ion batteries arise from the thermodynamic instability of zinc in water, leading to harmful by-products, surface passivation, and hydrogen evolution that degrade battery performance [116]. These processes severely degrade battery performance by increasing internal resistance, reducing active surface area, and promoting dendrite growth. To mitigate these effects, researchers have developed several effective strategies focused on electrolyte engineering, where neutral or mildly acidic electrolytes, such as zinc sulfate or zinc triflate, are used to reduce water activity and limit corrosive reactions [117,118]. The highly concentrated “water-in-salt” electrolytes further suppress corrosion by reducing the availability of free water molecules and altering the zinc ion solvation structure [119,120]. Additionally, functional additives, such as lithium salts, organic molecules like glucose, or surfactants, can form protective surface films, buffer local pH shifts, and inhibit the hydrogen evolution reaction [121,122,123]. Another important strategy involves constructing artificial interfacial layers on the zinc anode. Coatings made from materials like polyvinyl butyral or titanium dioxide act as physical barriers that prevent direct contact between water and zinc while maintaining ion conductivity, thus protecting the anode from corrosion and stabilizing zinc deposition [124]. Furthermore, gel polymer and solid-state electrolytes, which contain limited water and offer controlled ion transport, can reduce the occurrence of corrosive reactions and enhance the structural integrity of the electrode [125,126,127]. Finally, the use of zincophilic and corrosion-resistant substrates ensures more uniform zinc nucleation and deposition, minimizing localized corrosion and prolonging battery life [128].

In terms of application, Zn-ion batteries are particularly well-suited for large-scale energy storage in renewable energy systems, such as solar and wind power, and for stabilizing energy grids [129]. Other applications include powering IoT devices, providing backup power for homes and buildings, and potentially for EVs [130,131,132].

#### 3.3.2. LCA Zn-Batteries

Santos et al. [133] provide a foundational cradle-to-gate LCA of laboratory-scale Zn/air batteries, identifying key environmental hotspots, particularly cathode fabrication, and emphasizing the need for improved cyclability to achieve market competitiveness. Iturrondobeitia et al. [134] expand the analysis with a detailed LCA of six aqueous Zn-battery chemistries, quantifying 18 environmental impact categories and demonstrating that Zn-batteries can achieve competitive GWP and resource efficiencies relative to established technologies like Li-ion batteries. Meanwhile, Grignon et al. [135] take a broader systems-design perspective, proposing principles for developing organic cathodes tailored for grid-scale aqueous Zn-battery applications. The work emphasizes factors often overlooked in conventional LCA studies, such as cost per kWh, synthetic simplicity, and end-of-life biodegradability. Table 3 presents a comparative overview of the diverse approaches taken in the three studies by synthesizing and organizing key information, highlighting the main methodological frameworks, material focuses, and significant outcomes. This comparison aims to provide a clear understanding of how each study contributes to the environmental assessment of aqueous Zn-ion battery technologies.

Despite differences in scope, methodology, and material configurations, all studies converge on the view that Zn-based batteries present a promising alternative as post-Li battery technologies, particularly for stationary grid applications. Emphasizing cradle-to-gate impacts, material selection, energy use, and EoL considerations, the studies underscore the importance of early-stage environmental assessments in guiding future battery design and policy. Accordingly, the continued refinement of LCA methodologies and integration of recyclability, biodegradability, and cost-performance trade-offs will be crucial for advancing truly sustainable energy storage solutions.

### 3.4. Aluminum Battery Systems

#### 3.4.1. Mechanisms, Materials, and Interfacial Challenges

**Figure 7:**
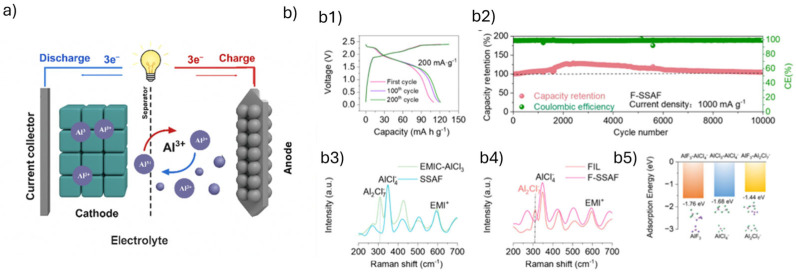
**a**) Schematic diagram illustrates the working principle of an Al-ion battery, **b**) electrochemical characterization and structure evolution of Al||C cells, **b1**) the discharge capacity retention, **b2**) of Al|F-SSAF|graphite cells, **b3**,**b4**) Raman spectra of different electrolytes, **b5**) comparison of the adsorption energy of ${AlF}_{3}-{AlCl}_{4}^{-}$, ${AlCl}_{3}-{AlCl}_{4}^{-}$, and ${AlF}_{3}-{AlF}_{3}{AlCl}_{4}^{-}$ published in reference [137] (Copyright © 2024. Published by American Chemical Society).

Al-ion batteries are emerging as promising contenders in the next generation of energy storage technologies, driven by the high volumetric capacity, cost-effectiveness, and abundant availability of aluminum [27]. The Al-ion working principle is based on either insertion or conversion mechanisms. In the insertion step, charge carriers such as Al^3+^ cations or complex chloroaluminate anions ([AlCl_4_]^−^ or [Al_2_Cl_7_]^−^) are reversibly inserted into host materials, often through a rocking-chair or dual-ion mechanism (Figure 7) [136].

Al-batteries have presented substantial advancements through the development of key materials: cathodes include transition metal oxides (e.g., MnO_2_, V_2_O_5_, TiO_2_), sulfides (e.g., NiS, MoS_2_, CoSe_2_), Prussian blue analogs, organic compounds, and various forms of carbon (graphite, graphene, CNTs) [138,139,140,141]. These materials are selected based on their ability to host Al^3+^ or [AlCl_4_]^−^ ions with high stability and capacity. The main advantages of cobalt sulfide (CoS_x_) cathodes in Al-ion batteries stem from their high theoretical capacity, good electrical conductivity, and chemical stability in aggressive electrolytes. These materials enable multiple charge storage mechanisms, including Al^3+^ intercalation and conversion to Al_2_S_3_, which allow for high energy density potential. Additionally, cobalt sulfides can be synthesized with nanostructured architectures (e.g., CoS_x_–CNT composites) that improve electron transport and surface accessibility, enhancing the kinetics of electrochemical reactions. Their compatibility with carbon matrices further boosts conductivity and mechanical integrity, making them attractive candidates for advanced cathodes despite the challenges that remain in achieving long-term stability and capacity retention [142].

The anode plays a pivotal role in the performance and stability of Al-ion batteries, and its development is closely tied to overcoming key challenges in both aqueous and non-aqueous systems [29]. The anodes are typically composed of aluminum metal, which offers high theoretical capacity but presents challenges such as dendrite formation, passivation, and hydrogen evolution in aqueous systems [143,144]. However, the presence of a naturally formed insulating Al_2_O_3_ layer on aluminum’s surface significantly hampers ion/electron transport, leading to poor plating/stripping efficiency [145,146]. In non-aqueous systems, this oxide layer can be progressively corroded by chloroaluminate anions, ultimately triggering dendrite formation and short-circuiting [147]. Innovative approaches like carbon-coated porous aluminum anodes and the use of weakly corrosive electrolytes (e.g., Aluminum trifluoromethanesulfonate (Al(OTF)_3_)/1-butyl-3-methylimidazolium trifluoromethanesulfonate ([BMIM][OTF]) have shown promise in mitigating these effects by enabling uniform ion deposition and improving cycle life [148,149].

The electrolyte composition and structure critically determine the battery’s electrochemical efficiency, longevity, safety, and commercial viability. In aqueous systems, challenges such as HER and high polarization voltages stem from the strong interaction between Al^3+^ ions and water, compounded by the inert Al_2_O_3_ layer [150]. Recent strategies, including surface modifications using non-aqueous electrolytes and pairing with eutectic electrolytes, have demonstrated notable improvements in stability, reversibility, and safety [151]. Ultimately, optimizing the aluminum anode through both surface engineering and electrolyte compatibility remains a critical frontier in advancing Al-ion technology. Wang et al. report the main advantage of considering anodes prepared with TiO_2_, which present the ability to eliminate the performance-degrading side reactions associated with metallic aluminum (e.g., as dendrite formation, passivation, and corrosion), thereby enabling a high-capacity, stable, and safe aqueous aluminum-ion battery configuration [152].

The electrolyte composition and structure critically determine the battery’s electrochemical efficiency, longevity, safety, and commercial viability. As discussed previously, in non-aqueous systems, electrolytes like chloroaluminate ionic liquids allow efficient electrochemical reactions but suffer from moisture sensitivity, high cost, and corrosiveness, limiting scalability and requiring inert conditions for fabrication [153,154]. In aqueous systems, electrolytes are more cost-effective and safer, but the strong interaction between Al^3+^ and water molecules triggers hydrogen evolution, corrosion, and anode passivation [155]. Innovations such as “water-in-salt” and eutectic electrolytes have significantly improved performance by modifying Al^3+^ solvation, suppressing hydrogen evolution, and enhancing cycling stability, all while offering broader temperature tolerance and reduced corrosion [151]. Guo et al. [137] successfully developed a recyclable solid-state aluminum-ion battery using an AlF_3_-assisted solid-state electrolyte, which significantly improved ion transport, suppressed aluminum corrosion, and enabled ultralong cycling stability of up to 10,000 cycles with high Coulombic efficiency. Their design also reduces the use of costly EMIC-AlCl_3_, enhancing safety and moisture resistance, thus introducing a sustainable approach by enabling >80% recyclability of the aluminum trifluoride (AlF_3_) framework.

Al-ion batteries exhibit unique advantages including high safety (nonflammable electrolytes), low environmental impact, and promising long-term cycling performance, with current and potential applications that span several sectors ranging from stationary energy storage (especially for stabilizing renewable energy sources like solar and wind), grid-scale storage systems (benefiting from their high volumetric capacity and cost-effective materials), and flexible and wearable electronics (where aqueous and solid-state Al-ion offer mechanical flexibility and safety) [156]. Looking ahead, improvements in cathode capacity, electrolyte compatibility, and interfacial stability will be key to bringing Al-ion batteries closer to commercialization [137].

#### 3.4.2. LCA Al-Batteries

Table 4 presents a chronological comparison of major LCA studies conducted on Al-ion batteries, which reflects the evolving understanding of Al-ion battery sustainability across various chemistries, system boundaries, and methodological approaches. From the earliest cradle-to-grave comparison with Li-ion batteries in 2019 to more recent work incorporating circular economy metrics and biobased materials, each study contributes unique insights into the environmental performance of Al-ion technologies. Table 4 highlights key differences in cell design, LCA scope, functional units used, and the dominant environmental impact drivers. It also captures how Al-ion batteries compare with conventional energy storage systems, emphasizing their potential advantages in terms of material sustainability, recyclability, and low toxicity, particularly in aqueous and bio-derived configurations.

Delgado et al. [157] conducted the first full cradle-to-grave LCA of a prototype Al-ion battery using an ionic liquid electrolyte (1-ethyl-3-methylimidazolium bis(trifluoromethylsulfonyl)imide ([EMIM[TFSI]): aluminum chloride (AlCl_3_) and a graphite cathode. Despite the low energy density of this early design (around 9 Wh kg^−1^), the study found that Al-ion batteries had a lower environmental impact per cell compared to Li-ion NMC batteries. However, due to the low energy output, their impact per Wh was higher, in which the main contributors to the environmental burden were found in the manufacturing phase, particularly from the synthesis of the ionic liquid and the high energy requirements of cell assembly.

Melzack et al. [158] shifted focus to an aqueous Al-ion battery using a TiO_2_ anode and a copper hexacyanoferrate (CuHCF) cathode. This cradle-to-gate LCA highlighted the significantly reduced environmental impacts of aqueous-based systems, especially when compared to supercapacitors. With a reported energy density of 15 Wh kg^−1^ and a cycle life of 1750 cycles, the cell demonstrated strong potential for low-impact, high-power applications. The key contributors to environmental impacts included the production of active materials and polymer binders, although the overall footprint was lower than that of comparable technologies. Building on this, Melzack [159] introduced a performance-based sustainability framework, using LCA to define environmental performance goals for emerging battery chemistries. The author used the concept of Competitive Functional Energy Density (CFED), while the study determined that the aqueous Al-ion battery would need to achieve at least 200.7 kWh kg^−1^ over its lifetime to match the GWP of conventional Li-ion batteries. This would require significant improvements in either energy density or cycle life, emphasizing the importance of LCA not just as a retrospective tool but as a design guide. Lastly, Mączka et al. [160] broadened the LCA landscape by analyzing several lab-scale Al-ion variants using different cathodes (WO_3_, Norit, and starch-derived CPS) and electrolytes (deep eutectic solvents and diethylene glycol). Their cradle-to-gate and end-of-life study incorporated circularity metrics and found that CPS + DEG cells had the lowest environmental impact. The use of bio-based materials and non-toxic, recyclable solvents positioned these designs as promising alternatives for sustainable energy storage.

Cooperatively, these studies illustrate the growing potential of Al-ion batteries as a sustainable alternative in the energy storage landscape. The early designs faced challenges such as low energy density and high-impact materials. Recent advancements, particularly the shift to aqueous electrolytes and bio-based components, demonstrate substantial reductions in environmental impact. The integration of LCA from early development stages, as seen in the performance-driven approach by Melzack [160], underscores the importance of aligning technological innovation with environmental objectives. As Al-ion technologies mature, future research should continue refining material choices, improving energy and cycle performance, and expanding end-of-life strategies to fully realize their role in low-impact, circular energy systems.

### 3.5. Metal–Air Batteries

#### 3.5.1. Anode Materials, Properties, and Performance Considerations

**Figure 8:**
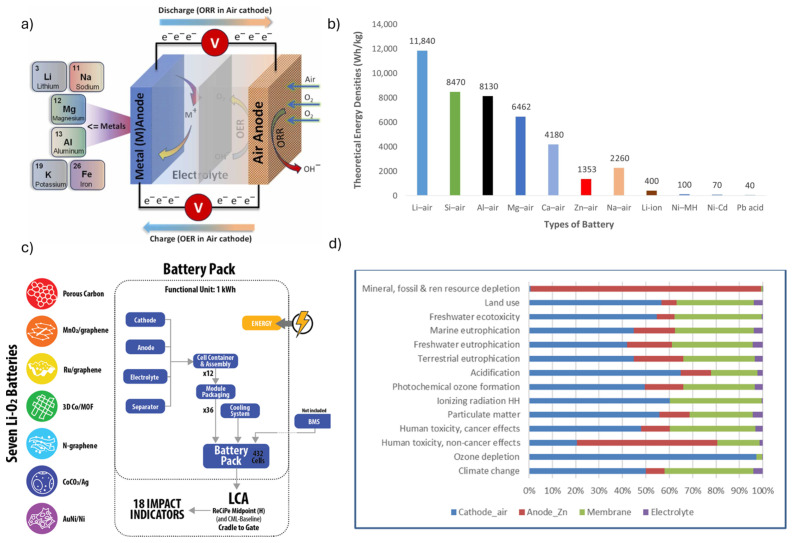
**a**) Scheme of metal–air batteries reproduced from reference [34] with Elsevier permission, **b**) Comparison of theoretical energy densities of metal-air and conventional batteries reproduced from reference [161] with permission from Elsevier, **c**) battery component scheme and LCA scope/boundaries for analyzed seven Li-O_2_ batteries (Copyright © CC-BY 4.0) and **d**) Zn/Air battery subassemblies to the fourteen impact categories analyzed (ILCD methodology) adapted from [133] with permission from Elsevier.

A metal–air battery is an electrochemical energy storage system that uses a metal (e.g., Li, Na, Zn, Al, or Mg) as the anode and ambient oxygen from the air as the cathode (Figure 8a). Unlike closed-cell batteries that store both electrodes internally, metal–air systems rely on oxygen supplied externally, which reduces cell weight and enables high theoretical energy density [34].

Metal-air battery configuration is attracting significant attention as a next-generation energy storage system, primarily due to its exceptionally high theoretical energy densities compared to conventional lithium-ion configurations (Figure 8b). To better illustrate the fundamental properties of all discussed configurations, Table 5 summarizes a comparison of Li, Na, Mg, Al, and Zn in terms of their valence, atomic weight, electrochemical potential, density, theoretical and volumetric capacities, as well as their natural abundance. This comparison highlights the trade-offs between high energy density, material cost, and practical performance, which ultimately guide the selection of suitable anode materials for different types of metal–air batteries.

From the analysis of Table 5, several insights emerge: the Li anode offers the highest theoretical gravimetric capacity (3862 mAh g^−1^) and the lowest electrochemical potential, making it ideal for lightweight, high-energy systems; however, its scarcity and reactivity are major limitations. In contrast, Na and Mg are far more abundant and inexpensive, but provide lower gravimetric capacities and face rechargeability issues. Al, despite its higher atomic weight, delivers extremely high volumetric capacity (8046 mAh cm^−3^) due to its trivalent nature, which is attractive for compact storage but complicated by stability problems. Zn, already widely used, combines moderate energy density with high safety and abundance, though its higher density reduces specific energy compared to Li or Al. Together, these trade-offs explain why Li dominates research interest, while Zn and Al remain practical candidates for commercial or large-scale deployment. By focusing on the anode’s intrinsic properties, the analysis remains valid across multiple configurations (air batteries, ion batteries, hybrid systems).

Li-air batteries have received the greatest focus within this configuration, due to Li-air systems being particularly notable for their extremely high theoretical energy density, positioning them as candidates for advanced electric vehicles, yet their complex reaction mechanisms and poor stability hinder near-term deployment [34,165]. Zn-air batteries, already used commercially in devices such as hearing aids and remote sensors, offer high energy density, cost-effectiveness, and safety advantages over Li-based systems, but face challenges such as limited cycle life and electrode degradation [166]. Al-air batteries stand out for their exceptionally high theoretical energy density (8.1 kWh kg^−1^), lightweight design, and recyclability, making them attractive for electric vehicles, military, and aerospace applications; however, issues of corrosion, hydrogen gas evolution, and non-rechargeability remain critical barriers [167]. Mg-air and sodium–air batteries also present promising attributes, such as low cost, material abundance, and environmental friendliness, but both suffer from limited rechargeability, passivation layer formation, and side reactions that reduce performance and durability [168,169].

#### 3.5.2. LCA Metal–Air Batteries

Despite their promising energy density and material advantages, metal–air batteries remain relatively underexplored from an LCA perspective. Most existing studies are limited to Li-air/ion configurations [170], often at laboratory or prototype scale, which restricts their ability to capture the environmental impacts of scaled-up manufacturing and real-world operation [165]. Critical hotspots such as cathode material synthesis, passivation phenomena, and sensitivity to air purity are insufficiently represented in current inventories, leading to uncertainties in comparative results. The work performed by Zackrisson et al. [171] demonstrated that, even at prototype scale, production processes dominated the environmental profile, with copper use driving toxicity-related indicators and electricity consumption driving GWP. More recent studies have expanded this perspective, exploring a broader range of lithium-oxygen chemistries and production pathways. Iturrondobeitia et al. [172], for example, compared seven lithium-oxygen variants with Li-ion, Li-S, and Na-ion benchmarks, finding that five of the seven chemistries delivered lower greenhouse gas emissions per unit energy capacity than the lowest-impact Li-ion reference. Yet, the study also highlighted several trade-offs: cobalt carbonate- and gold/nickel-based variants exhibited substantially higher emissions, underscoring the sensitivity of results to cathode composition. Early assessments of Al-air systems were largely cost-focused, with limited environmental detail [173]. More recently, Santos et al. [133] examined Zn-air systems at laboratory scale, identifying cathode production as the dominant contributor to most impact categories, while the Zn anode drove human toxicity and resource depletion indicators. Interestingly, this study also reported competitive costs on a per-power capacity basis, suggesting that Zn-air systems may be attractive for high-power stationary applications, though energy-normalized costs remain comparatively high.

### 3.6. Methodological Standards in Battery LCA

Material structures strongly affect recovery efficiency, especially in emerging chemistries. For example, while Na-based Prussian blue analogs offer open frameworks favorable for leaching, their vacancy defects and moisture sensitivity reduce purity. In multivalent systems, Mg cathodes (e.g., Chevrel phases) and Zn-based MnO_2_ polymorphs often degrade into complex byproducts, while Al-ion batteries rely on corrosive electrolytes that complicate processing. As a result, universal recycling strategies remain limited, and recovery processes must be adapted to each chemistry, though common design-for-recycling principles can still guide more sustainable solutions [174]. Across Na-, Mg-, Zn-, and Al-based batteries, the evidence base is still uneven, but the pattern is clear: circularity choices made at design time (modularity and easy disassembly, recyclable/benign materials, and standard EoL routes) shift impacts away from manufacturing hotspots and enable large, avoided burdens at end-of-life. Embedding circularity principles in battery design strengthens the link between technical performance and life-cycle sustainability. As an example of this strategy, Tian et al. [175] achieved 85% retention over 1000 cycles using necklace-like B,N,F-doped carbon fibers with anchored Sn nanoparticles to stabilize anode-free Li deposition, reducing material inputs and easing end-of-life recovery. Similarly, An et al. [109] demonstrated a Na-MXene@Sn heterointerface enabling dendrite-free, reversible Zn plating/stripping with ~99.5% Coulombic efficiency over 5000 mAh cm^−2^, enhancing energy density while supporting recycling and circular value chains.

Building on these recycling challenges, recent years have also seen LCA evolve beyond material recovery concerns toward a holistic, forward-looking approach spanning the entire battery value chain. Additionally, establishing quantitative links between electrochemical degradation pathways and life cycle impacts remains methodologically complex but critical, as reduced cycle life inflates environmental burdens per FU (e.g., kWh delivered). Empirical LCA on Mg and Zn systems demonstrates that dendrite growth, corrosion, and hydrogen evolution diminish their relative resource and climate advantages by lowering lifetime energy throughput. Integrating battery material degradation/aging kinetics into pLCA or dynamic LCA frameworks would offer a pathway to transform failure mechanisms into lifetime-normalized sustainability metrics [176].

Over the past five years, LCA practice has moved from a handful of retrospective studies on Li-ion cells toward a much broader, forward-looking discipline that now covers the entire electromobility value chain, including cell chemistry choice, supply-chain localization, second-life use, and EoL routes [177]. Recycling and EoL strategies form essential components of sustainable battery management; nevertheless, they partially capture the environmental footprint associated with energy storage systems [178,179]. Methodologically, conventional process-based LCA is being complemented by pLCA and dynamic LCA, which integrate learning curves, grid-decarbonization pathways, and future collection rates to assess technologies that have not yet reached commercial scale [180,181]. From Li-ion batteries’ perspective, the environmental “hot-spots” have been related to cathode-grade precursor production and cell formation account for 35–60% of cradle-to-gate GHG emissions for state-of-the-art Ni, Mn, Co precursors, while recycling is expected to cut those burdens by ≥58% when hydrometallurgical or direct-recycling routes are substituted for virgin mining and refining [182]. LCA of any energy storage device, particularly batteries, poses several significant challenges that stem largely from the complexity and variability of battery systems, their applications, and the data requirements involved [183].

**Figure 9:**
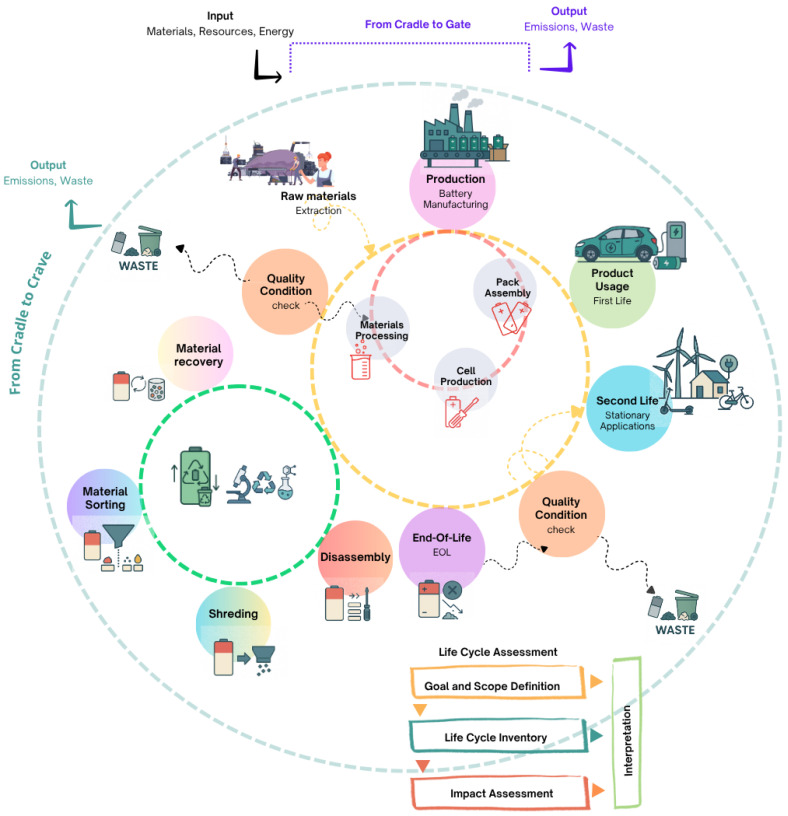
Overview of the battery LCA stages. Color codes are used to group related stages of the battery life cycle, distinguishing production, usage, end-of-life, and recovery processes for clarity.

Figure 9 provides the journey of a battery beginning with the extraction of raw materials and continuing through production (materials processing, cell production, and pack assembly), first use in, for example, an EV, potential second-life applications in stationary energy storage ensured by a quality check, and ultimately to its EOL phase. It further illustrates the processes involved in battery recycling, which may include disassembly, material sorting, shredding, and recovery, highlighting points at which waste is generated or materials are reintegrated into the production cycle. The diagram also emphasizes the key components of LCA, which include goal and scope definition, life cycle inventory, impact assessment, and ultimately interpretation. One of the foremost difficulties is defining a consistent and comprehensive assessment framework, since LCA of batteries often varies in scope, methodology, and level of detail, which undermines the comparability and policy relevance of results [184].

Table 6 operationalizes the systems diagram by mapping each discrete node in the battery value chain based on Figure 8 dynamics to (i) its principal sources of methodological and empirical complexity and (ii) the corresponding gaps in the peer-reviewed and industrial literature. The central column identifies dominant complexity drivers ranging from volatile critical-metal supply dynamics to state-of-health diagnostic uncertainty, while the right-hand column specifies where current LCA, techno-economic analysis, and circular-economy modeling either diverge methodologically or lack statistically robust datasets.

In a very recent work, Peters proposed that to enhance the relevance, comparability, and policy value of battery LCAs, the studies must follow a tiered framework of best practices emphasizing transparent inventory data, consistent system boundaries, appropriate functional units, consideration of evolving energy mixes, applications, and recycling processes [195]. To be meaningful, a study must clearly define the application context, including charge profiles, charge/discharge rates, and environmental conditions, as these parameters critically affect battery lifetime and efficiency. Moreover, the FU (e.g., per kWh of discharged electricity over the battery’s life) must be carefully chosen to ensure comparability across studies and technologies. Another persistent challenge lies in the data quality and availability for LCA inventory, since the existing models must consider the electrochemical properties of materials, energy consumption in manufacturing, and the origin/quality of raw and secondary materials [196,197]. This data is frequently incomplete, outdated, or confidential, complicating the reproducibility and robustness of studies. Song et al. [198] applied CiteSpace bibliometric analysis to map and synthesize LCA research across diverse power battery technologies (e.g., Li-ion, fuel cell, lead–acid), integrating environmental impacts, recycling strategies, and policy frameworks; pinpointing critical gaps in system boundary alignment, primary/localized data, database coverage; and charting focused future directions, such as cost assessment, circular economy integration, and resource-use modeling to guide more sustainable battery development. The usage phase is particularly problematic due to its heterogeneity with factors related to consumption, self-discharge, and round-trip efficiency, which may vary widely depending on the battery’s application and operational profile [199]. These variables can have a significant impact on the overall environmental performance but are difficult to model accurately without detailed real-world data, which is often lacking. Additionally, modeling battery lifetime remains a core challenge due to degradation processes that are governed by complex mechanisms influenced by C-rate, depth of discharge, temperature, and other stress factors [200]. LCA reliability, general lifetime data for different battery types and applications are still scarce, and few studies adequately model these dynamics, which introduces major uncertainty into results [198]. EOL stage also introduces uncertainty since the recycling potential of batteries is increasingly recognized; nevertheless, current data on recycling processes and material recovery rates are still limited and often speculative [201]. Lastly, the impact of electricity used in the whole value chain adds further complexity with ongoing energy transition efforts involving the decarbonization of the grid. Ideally, LCA studies should use time- and location-specific electricity data and consider how battery operation aligns with grid dynamics. However, such detailed modeling is rarely feasible in standard assessments [195].

LCA is already a standardized methodology under frameworks like ISO 14040/44 [1,202]; nevertheless, the application to batteries requires additional refinement due to their complex behavior, diverse battery chemistry materials, and context-dependent performance [177]. A central recommendation is the use of a common assessment framework that includes all relevant life cycle stages, production, use, and EOL. Eltohamy et al. [203] highlight that current LCA for EV batteries are highly inconsistent, especially in data quality, system boundaries, and electricity modeling, underscoring the urgent need for harmonized methods to improve transparency, comparability, and decision-making in battery sustainability evaluations. The future studies must clearly define their system boundaries, the level of analysis (e.g., cell, pack, or system), and whether they follow an attributional (descriptive) or consequential (decision-based) approach. This foundational clarity ensures meaningful comparisons between different technologies and applications. Another key methodological standard is the selection of an appropriate FU, typically based on service provided, such as 1 kWh of discharged energy or 1 km driven (in the case of electric vehicle batteries) [204]. This enables performance-based assessments that reflect real-world usage while allowing results to be rescaled and compared across studies. Standardization also extends to modeling the EoL stage, which remains a major uncertainty in battery LCA [205,206]. The current regulatory frameworks are primarily tailored to Li-ion batteries and lack specific guidance for emerging chemistries, creating a gap that is expected to be addressed through updated policies and standards for emerging technologies [199].

Accordingly, to make LCAs across post-Li chemistries directly comparable, this review highlights the importance and need for a standardized, chemistry-agnostic framework with: (i) common goal/scope rules, (ii) harmonized FU tied to service delivered, (iii) aligned system boundaries that include second life and EoL, and (iv) transparent data-quality and reporting checklists. All this is built on ISO 14040/44 and recent best practice calls specific to batteries. In particular, i) the level of analysis must be explicit (cell/module/pack/system) and consistent within comparisons. A system boundary stratification (to more easily be applied uniformly across chemistries) uses a minimum cradle-to-grave boundary that includes raw materials, cell/pack manufacturing, use, quality-check and routing, second-life (if applicable), and EoL (collection, disassembly, sorting, recycling/valorization, disposal), as it was attempted in the table mapping processes to data sources. In point ii), the establishment of a harmonized FU anchored to a “delivered service” (e.g., pair the FU with performance-normalization parameters useful life, cycle count to EoL criterion, capacity retention, efficiency). Regarding points iii) and iv), a minimum reporting checklist to ensure reproducibility and comparability to each study should, at minimum, provide: (a) application and analysis level; LCA type; (b) FU(s) + standard duty cycle parameters and lifetime/EoL criteria; (c) system boundary diagram and process-to-data mapping table; (d) electricity mixes, transport distances, and manufacturing energy; (e) detailed list of all the materials, components, and quantities required to manufacture a battery cell, module, or pack with data-quality scores and uncertainty ranges; explicit gaps; (f) recycling scenarios and allocation rules; material recovery rates; and (g) full sensitivity/uncertainty analysis (lifetime, efficiency, electricity mix, recovery). Finally, hosting inventories and metadata in open-access repositories and connecting to digital battery passports to track sourcing, manufacturing energy, and EoL outcomes is expected to bring improvements in the LCA comparability across battery emerging chemistries over time.

The adoption of a unified and comparable LCA framework is expected to evolve gradually: in the near term, efforts must focus on building consensus around core principles such as standardized FU, system boundaries, and transparent reporting requirements, supported by open data templates and pilot studies to test feasibility. Over the medium term, broader adoption by researchers, industry, and policy initiatives will drive harmonization, integration with digital tools, and the use of prospective modeling as data availability improves; while in the longer term, these practices are likely to be formalized through international standards and regulatory frameworks, enabling consistent, transparent, and policy-relevant environmental assessments across emerging technologies.

### 3.7. Critical Resources, Battery Production, and Life Cycle Implications

As battery manufacturing depends heavily on critical materials like copper, aluminum, and nickel, the stability and availability of these materials are vital. Europe’s import demand for these energy transition minerals has continued to rise in tandem with its shift toward solar and wind energy despite mounting financial stress, price volatility, and geopolitical uncertainties. For battery production, access to core inputs can become uncertain precisely when demand for critical materials is high. Conversely, Mariev and Islam [207] study finds that sound political and electoral governance structures can help safeguard mineral imports by promoting stable trade environments. However, geopolitical events such as the Russia-Ukraine conflict have introduced new layers of volatility, revealing how vulnerable the critical materials supply chain can be to external shocks [208]. This has direct implications for the battery sector, where uninterrupted access to key materials is essential for scaling production of emerging technologies. The findings support the urgency behind policy actions like the EU’s Critical Raw Materials Act, which aims to build more resilient and diversified supply chains. LCA depends heavily on accurate, up-to-date data about battery critical raw material flows, trade routes, and sourcing regions [2]. Financial stress, price volatility, and geopolitical risks can cause supply chain disruptions or shifts in sourcing countries, making it difficult for battery LCA practitioners to accurately model supply chains over time while maintaining consistent assumptions about sourcing (e.g., will nickel come from Russia, Indonesia, or Finland?) [209]. Static LCA databases may not capture dynamic geopolitical or economic/market-driven shifts, resulting in outdated or unrealistic assumptions.

To address these challenges, a forward-looking approach to battery supply chain assessment must integrate geopolitical risk analysis, real-time trade data, and scenario-based modeling into Life LCA frameworks. This would enable more resilient and adaptive sustainability evaluations, helping industry actors and policymakers anticipate disruptions and adjust sourcing strategies accordingly. Furthermore, fostering international cooperation, investing in domestic extraction and refining capacities, and enhancing material circularity through recycling and reuse will be crucial for reducing dependency on unstable or concentrated supply chains. As Europe continues its transition to clean energy and battery-based technologies, aligning policy frameworks like the Critical Raw Materials Act with evolving LCA methodologies will be essential to ensure both the environmental integrity and strategic security of its energy systems.

Overall, more rigorous methodological standards must be applied consistently to battery LCA that are scientifically robust, comparable, and actionable for policymakers and industry stakeholders. These standards must provide a path toward harmonizing practices and improving the credibility and utility of sustainability assessments in the rapidly evolving battery sector and emerging energy storage technologies.

## 4. Conclusions

Looking ahead, advancing the sustainability of emerging battery chemistries such as sodium-, magnesium-, zinc-, and aluminum-based systems will require a multifaceted approach that integrates methodological, technological, and policy innovations. A key priority lies in the standardization of LCA methodologies, particularly in establishing consistent system boundaries, FU, and data quality criteria that can accommodate the unique characteristics of each chemistry. Harmonized frameworks and open-access repositories will be essential to enable robust, transparent comparisons across technologies. Equally important is the integration of circular economy principles early in battery design, emphasizing recyclability, modularity, and the use of non-toxic, easily recoverable materials. Scalable recycling processes, such as direct and electro-/chemical recovery, must be developed in parallel with battery innovation, supported by industrial-scale demonstrations and tailored to chemistry-specific challenges. Second-life applications offer promising avenues for extending battery value and reducing environmental burdens, provided that clear diagnostic standards and digital battery passports are established to manage aging and EoL logistics effectively. Moreover, regulatory alignment with evolving policies such as the EU Battery Passport and extended producer responsibility will play a crucial role in shaping sustainable battery ecosystems. To capture future developments, prospective and dynamic LCA models should be more widely adopted, integrating projections of grid decarbonization, material learning curves, and evolving usage profiles. These models must be underpinned by improved data availability, particularly for less-studied chemistries like Mg- and Al-ion systems, which currently suffer from limited real-world datasets. Finally, interdisciplinary collaboration between researchers, policymakers, and industry stakeholders will be critical to developing meaningful sustainability benchmarks and ensuring that new battery technologies are not only technically viable but also environmentally and socially responsible.

## Figures and Tables

**Table 1:** Overview of Environmental Impact Indicators commonly used in battery LCA [2].

	Midpoint Category	Definition	Abrev. *	Unit
**Climate and Atmospheric Impacts**	Global warming	Measures greenhouse gas emissions contributing to climate change, expressed as CO_2_-equivalent over 100 years.	GWP	kg CO_2_ eq
	Stratospheric ozone depletion	Assesses emissions of substances that deplete the stratospheric ozone layer.	ODP	kg CFC11 eq
	Ionizing radiation	Quantifies radioactive emissions that can impact human health and ecosystems.	IRP	kBq Co-60 eq
	Ozone formation (Human health)	Estimates emissions of ozone precursors (e.g., NO_x_, VOCs) harmful to human health at the ground level.	EOFP	kg NO_x_ eq
	Ozone formation (Terrestrial ecosystems)	Assesses emissions of ozone precursors impacting terrestrial ecosystems.	POCP	kg NO_x_ eq
**Air Pollution and Human Health**	Human carcinogenic toxicity	Evaluates emissions of substances with the potential to cause cancer in humans.	HTP-c	kg 1,4-DCB eq
	Human non-carcinogenic toxicity	Assesses substances harmful to human health without causing cancer.	HTP-nc	kg 1,4-DCB eq
	Fine particulate matter formation	Measures emissions leading to the formation of fine particles (PM_2.5_), affecting air quality and human health.	PMFP	kg PM_2.5_ eq
**Resource Use**	Water consumption	Measures the total amount of freshwater consumed throughout the life cycle.	WSF/ WDP	m^3^
	Mineral resource scarcity	Quantifies depletion of mineral resources.	ADP_m_	kg Cu eq
	Fossil resource scarcity	Measures depletion of fossil fuel resources.	ADP_f_	kg oil eq
**Ecosystem Impacts**	Freshwater eutrophication	Measures phosphorus emissions causing nutrient enrichment and algal blooms in freshwater bodies.	FEP	kg P eq
	Marine eutrophication	Assesses nitrogen emissions leading to over-fertilization in marine environments.	MEP	kg N eq
	Terrestrial ecotoxicity	Evaluates the toxic effects of chemical emissions on terrestrial ecosystems.	ETP-t	kg 1,4-DCB eq
	Freshwater ecotoxicity	Quantify emissions of toxic substances affecting freshwater ecosystems.	ETP-fw	kg 1,4-DCB eq
	Marine ecotoxicity	Measures the impact of toxic substances on marine ecosystems.	ETP-m	kg 1,4-DCB eq
	Land use	Assesses impacts of land occupation or transformation on ecosystems, expressed in area × time.	-	m^2^ a crop eq
	Terrestrial acidification	Quantifies emissions (e.g., SO_2_, NO_x_) that lead to acid deposition in soils and freshwater, affecting biodiversity.	AP	kg SO_2_ eq

* The abbreviation of LCA midpoint indicators is often challenging due to inconsistencies across different impact assessment methods, where overlapping terms and varying nomenclature can lead to ambiguity and hinder direct comparison between studies.

**Table 2:** Structured LCA overview of Na-Ion battery technologies.

LCA Phase	Subcategory	Wickerts et al. (2024) [64]	Batuecas et al. (2024) [65]	Zhang et al. (2024) [66]	Guo et al. (2023) [67]	Mozaffarpour et al. (2022) [68]
**1. Goal and Scope**	Goal	pLCA for large-scale Na-ion batteries	Solid/liquid Na/Li batteries at lab scale	GHG impacts of Na-ion batteries	LCA of Na-ion vs. lithium iron phosphate technology for EVs	Evaluate cathode synthesis routes
	System Boundary	Cradle-to-gate	Cradle-to-gate	Cradle-to-grave	Cradle-to-grave	Cradle-to-gate
	FU	1 kWh capacity	1 kWh capacity	1 kWh of energy delivered	1 kWh, and the total mileage (200,000 km)	1 kWh capacity
**2. Inventory Analysis**	Battery composition	Anode: HC from phenolic resin, HC from lignin. Cathode: Prussian white Electrolyte NaPF_6_ in EC/DEC (1:1), NaBOB in triethyl phosphate	Electrolyte NASICON Anode: Na Cathode: Sodium Iron Phosphate, Polyvinylidene fluoride, Carbon black, Aluminum foil	NaPBA Prussian blue analogs. Various Na cathodes, HC	Nickel-based cathode materials. Aluminum foil for electrodes. High energy input (electricity) for cathode production. NMP as a solvent in electrode fabrication.	(3 routes) Na_3_MnCO_3_PO_4_ (NMCP) cathode
	Energy Source	Grid-mix + fossil-free options	Concentrating solar power (CSP) is the best case	Future decarbonized grids	China’s electricity mix	Iranian electricity
	Manufacturing Process	Based on the Li-batteries gigafactory model	Lab-scale modeling	Dimensional model for 21,700 cells	General EV battery production	Lab-scale synthesis methods
**3. Impact Assessment**	Impact Assessment Methods	ReCiPe 2016 Ecoinvent database (version 3.8)	CML (Centre of Environmental Science, Leiden University)	pLCA combining future scenario modeling with integrated assessment models (IAMs)	Ecoinvent 3.7.1 database, employing SimaPro software ReCiPe 2016 (H) midpoint method	Ecoinvent v3.0 database, SimaPro 8.3 software
	GWP	~On par with Li technology batteries	Higher than Li batteries unless CSP is used	↓ 43–57% from 2020 to 2050	Slightly worse than LFP LIB	14–20 kg CO_2_/kg NMCP
	ADPₘ	Significantly lower than Li batteries	Reduced due to no Li/Co/Ni	Emphasized via sodium abundance	Nickel-based active materials	Reduced with ball milling
	Human Toxicity	Lower with lignin anodes	Lowest for solid SIBs	Lower toxicity in the long term	Li batteries are better in some impact categories	Not assessed
	EP/POCP	Lower than Li batteries overall	Worse for ozone/eutroph. vs. liquid Li-ion battery	No details	Li batteries are better in eutrophication	The ball milling route is better
	Energy Demand	Sensitive to the anode and the electricity source	Solid SIB w/CSP lowers CED significantly	Grid decarbonization is key	Production phase dominates	Electricity-intensive methods
**4. Interpretation**	Key Findings	Lignin HC and green electricity	Solid Na-batteries are promising if optimized	Na-batteries become climate-competitive by 2050	Na-ion is better in the long term	Ball milling preferred
	Trade-Offs Identified	Electrolyte uncertainty	Ozone depletion trade-off	Manufacturing ↓, material ↑ in impact share	Na battery wins with reuse or recycling	Acidification ↑ for hydrothermal methods
	Limitations	Prospective modeling assumptions	Lab-scale not scaled to industry	Future projections have uncertainty	Simplified recycling scenarios	Only cradle-to-gate considered
	Recommendations	Green power + bio-HC	Dematerialize electrolyte + renewable energy	Invest in cathode efficiency and a decarbonized grid	Use gradient utilization + recycling	Prefer ball milling synthesis

Note: The arrow ↓ indicates a decrease or reduction in that value, ↑ indicates an increase or rise.

**Table 3:** Comparative analysis of LCA outcomes for aqueous Zn-Ion battery technologies.

Parameter	Santos (2020) [133]	Iturrondobeitia et al. (2022) [134]	Grignon et al. (2022) [135]
**Goal of Study**	Cradle-to-gate system boundary. Assessment of circularity and sustainability	Cradle-to-gate environmental impact of six AZIB chemistries	Design principles for sustainable organic cathodes
**Battery Focus**	Aqueous Zn-batteries	6 lab-scale aqueous Zn-batteries with varying cathode types	Organic cathodes for grid-scale aqueous Zn-batteries
**Functional Unit**	1 kWh of stored energy, sometimes extended to 1 kWh of “lifetime” energy storage, when an average of all cycles’ capacity until the end of life is considered	1 kWh of energy storage	Cost and material design per $/kWh
**LCA Methodology**	Material Circularity Indicator, Ecoinvent Swiss database, software SimaPro	ReCiPe 2016 Midpoint, Ecoinvent 3.7, OpenLCA	Life cycle thinking (qualitative)
**System Boundary**	Cradle-to-gate/gate-to-grave variants	Cradle-to-gate (manufacturing phase only)	Focus on synthesis and end-of-life scenarios
**Environmental Indicators**	GHG emissions, resource use, circularity	18 indicators incl. GWP, toxicity, eutrophication, etc.	GWP, biodegradability, and synthetic scalability
**Key Findings**	Circularity is often not linked to lower impacts	Zn-based chemistries are competitive with Li-batteries/Na-technologies	Organic cathodes can reduce the environmental burden
**Material Highlights**	Zn, MnO_2_, organic options	Co_3_O_4_, V_2_O_5_, Na_3_V_2_(PO_4_)_3_, Prussian Blue, MoS_2_, CALIX4-C4Q	Pyrene-4,5,9,10-tetraone, poly(anthraquinonyl sulfide)
**Electrolyte Type**	Aqueous vs. organic electrolyte comparison	Aqueous electrolytes, Zn-based salts	Emphasis on non-corrosive, low-cost aqueous electrolytes
**Energy Density Range**	Moderate to low for aqueous Zn-batteries	100–361 Wh kg^−1^ across chemistries	Targeting practical areal loading (e.g., 5 mAh cm^−2^)
**End-of-Life Considerations**	Circular economic indicators	Recycling not included (early-stage tech)	Focus on biodegradability, safe disposal pathways

**Table 4:** Comparative LCA of Al-Ion battery technologies—a chronological overview (2019–2024).

Ref.	Cell Chemistry/Design	LCA Scope	Functional Unit	Major Contributors to Impact	Key Environmental Outcomes	Comparison vs. Li-Ion/Other Tech	Highlight
**Delgado et al. (2019)** **[157]**	Al-anode, graphite cathode, [EMIM][TFSI]:AlCl_3_ electrolyte (18650 format)	Cradle-to-grave	Per-cell manufactured and per-Wh of storage capacity	Manufacturing phase (especially ionic liquid production and energy use)	Lower GWP per cell than Li-ion NMC; higher per Wh due to low energy density	Li-ion is more efficient per Wh; Al-ion is favorable per cell	First full-process Al-ion LCA; uses dual FU; TRL still low
**Melzack et al. (2021)** **[158]**	Copper hexacyanoferrate (CuHCF) cathode/TiO_2_ anode; aqueous AlCl_3_ + KCl electrolyte	Cradle-to-gate	Per kWh (is defined as the total amount of energy given over a lifetime (per kg)	Electrode and electrolyte synthesis carbon–polymer substrate	Lower impact than supercapacitors; low toxicity, aqueous safe chemistry	Competitive with supercapacitors in GWP and resource use	Introduces hybrid-cell comparison; uses OpenLCA modeling
**Melzack (2022)** **[159]**	Same aqueous Al-ion (CuHCF/TiO_2_)	Cradle-to-gate	Functional Energy Density (kWh/kg over life)	Active material % life cycle limitations	Current: 26.5 kWh kg^−1^; needs ≥200.7 kWh kg^−1^ to match Li-ion GWP	Requires ~14,000 cycles or improved energy/capacity ratio	Proposes CFED metric; links design and sustainability targets
**Mączka et al. (2024)** **[160]**	Multiple lab-scale variants	Cradle-to-gate (production + EoL)	Per cell (normalized to 100 F g^−1^ capacitance)	Electricity usage (lab-scale Polish grid); cathode/electrolyte choice	Best performer: CPS + DEG; DES variants are also favorable	Significantly lower EoL impact than Li-ion	First LCA with circularity indicators, uses biobased cathodes and green solvents

**Table 5:** Comparison of Li, Na, Mg, Al, and Zn anodes.

Element	Li	Na	Mg	Al	Zn
**Valence**	+1	+1	+2	+3	+2
**Atomic weight**	6.94	22.99	24.31	26.98	65.38
**Potential/V (vs. SHE)**	−3.04	−2.71	−2.36	−1.68	−0.76
**Density/g cm^−3^**	0.534	0.968	1.738	2.70	7.14
**Theoretical capacity/mAh g^−1^**	3862	1166	2205	2980	820
**Volumetric capacity/mAh cm^−3^**	2062	1128	3832	8046	5854
**Anode Cost/USD kg^−1^**	>250	2.7	2.5	1.9	2.0
**Abundance/wt.%**	0.0018	2.27	2.30	8.20	0.0070
**Ref.**	[162]	[162]	[162]	[163]	[164]

**Table 6:** Complexity drivers and literature gaps across the battery life cycle.

Zone in Figure 8	What Makes it Complex?	Where the Literature is Thin (or Contradictory)	Ref.
**Raw-materials extraction**	Volatile critical-metal markets, opaque social impacts, rapidly changing chemistries	Dynamic, region-specific inventories of future mining vs. recycled supply; social-LCA datasets for cobalt, nickel, Li; coupling geology with geopolitical risk models	[2,182]
**Cell and pack manufacturing** (dashed red loop)	High scrap rates, heterogeneity of chemistries, and fast process innovation	Real-time scrap quantification and its fate; pLCA for solid-state/Na-ion lines; allocation rules when closed-loop recycling feeds back into cell production	[177,185]
**First-life use (EV)**	Degradation strongly depends on driving behavior, climate, and charging profile	Global-scale, high-resolution duty-cycle datasets; physically based aging models validated beyond 5–8 yrs; incorporation of user behavior in LCA	[186,187]
**Quality-condition checkpoints**	State of health/state of charge (SOH/SOC), measurement uncertainty, lack of a universal “battery passport”	Standardized diagnostics that work across chemistries and form factors; digital-twin integration with BMS and EoL routing	[188]
**Second-life deployment**	Diverse stationary load profiles, financing risk, and regulatory ambiguity	Techno-economic models that co-optimize capacity fade, revenue stacking, and policy incentives; methods for allocating environmental credit between 1st- and 2nd-life	[189,190]
**End-of-Life (EOL) and reverse logistics**	Unknown battery chemistries, safety hazards, and fragmented ownership	Geo-spatial models of EOL-flows, robust disassembly standards, and design-for-disassembly metrics in early design stages	[190]
**Shredding/material sorting**	Sensor-based sorting must identify chemistry, SOC, and embedded components	Open-access performance data for sensor suites; AI-enabled real-time sorting algorithms	[191]
**Material-recovery pathways** (hydro-, pyro-, direct recycling)	Trade-off between purity, energy demand, and capex; scaling lab success to industrial reality	Plant-level LCI inventories for direct-recycling; techno-economic learning curves; comparative studies that include low-value chemistries	[192,193,194]
**LCA block (bottom-right)**	Need for dynamic, circular LCAs that track recursive loops and time-variant grids	Harmonized functional units for second-life, temporal differentiation of impacts, integration with material flow analysis	[2,177]

## Data Availability

No new data were created or analyzed in this study. Data sharing is not applicable to this article.

## Abbreviations

The following abbreviations are used in this manuscript:

CATL	Contemporary Amperex Technology Co., Limited company
CNT	Carbon nanotubes
EoL	End-of-life
EU	European Union
EV	Electric Vehicle
FU	Functional Unit
ISO	International Standard of Organization
GHG	Greenhouse Gases
GWP	Global Warming Potential
HC	Hard Carbon
HER	Hydrogen Evolution Reaction
LCA	Life Cycle Assessment
LCI	Life Cycle Inventory
MOF	Metal–Organic Frameworks
NaBOB	Sodium Bis(oxalato)borate
NASICON	Sodium super ionic conductor (Na_1+x_Zr_2_SixP_3−x_O_12_, 0 < x < 3)
NMC	nickel-manganese-cobalt batteries
NVP	Sodium Vanadium Phosphate Na_3_V_2_(PO_4_)_3_
pLCA	Life Cycle Assessment
OER	Oxygen Evolution Reaction
ORR	Oxygen Reduction Reaction
PBA	Prussian blue analogs
SOC	State of charge
SOH	State of health
TRL	Technology Readiness Level

## References

[1] (2006). In: Environmental Management–Life Cycle Assessment–Principles and Framework.

[2] Paul D., Pechancová V., Saha N., Pavelková D., Saha N., Motiei M., Jamatia T., Chaudhuri M., Ivanichen-ko A., Venher M. (2024). Life cycle assessment of lithium-based batteries: Review of sustainability dimensions. Renew. Sustain. Energy Rev..

[3] Phogat P., Dey S., Wan M. (2025). Comprehensive review of Sodium-Ion Batteries: Principles, Materials, Performance, Challenges, and future Perspectives. Mater. Sci. Eng. B.

[4] Wu Y., Shuang W., Wang Y., Chen F., Tang S., Wu X.-L., Bai Z., Yang L., Zhang J. (2024). Recent Progress in Sodium-Ion Batteries: Advanced Materials, Reaction Mechanisms and Energy Applications. Electrochem. Energy Rev..

[5] Zhao L., Zhang T., Li W., Li T., Zhang L., Zhang X., Wang Z. (2023). Engineering of Sodium-Ion Batteries: Opportunities and Challenges. Engineering.

[6] Bača P., Libich J., Gazdošová S., Polkorab J. (2025). Sodium-Ion Batteries: Applications and Properties. Batteries.

[7] Kim H. (2023). Sodium-Ion Battery: Can It Compete with Li-Ion?. ACS Mater. Au.

[8] Carter R., Waller G., Jacob C., Hayman D., West P., Love C. (2025). First Look at Safety and Performance Evaluation of Commercial Sodium-Ion Batteries. Energies.

[9] Gao Y., Zhang H., Peng J., Li L., Xiao Y., Li L., Liu Y., Qiao Y., Chou S.-L. (2024). A 30-year overview of sodium-ion batteries. Carbon. Energy.

[10] Dorau F., Sommer A., Koloch J., Röß-Ohlenroth R., Schreiber M., Neuner M., Abo Gamra K., Lin Y., Schöberl J., Bilfinger P. (2024). Comprehensive Analysis of Commercial Sodium-Ion Batteries: Structural and Electrochemical Insights. J. Electrochem. Soc..

[11] Ye X., Xiao X., Wu Z., Zhan Y., Wu X., Liu S. (2024). Recent advances in rechargeable aqueous magnesium-ion batteries. J. Mater. Chem. A.

[12] Qu X., Li G., Wang F., Zhang Y., Gao T., Luo Y., Song Y., Fang F., Sun D., Wang F. (2025). In-situ electrochemical activation accelerates the magnesium-ion storage. Nat. Commun..

[13] Setiawan D., Lee H., Pyun J., Nimkar A., Shpigel N., Sharon D., Hong S.-T., Aurbach D., Chae M.S. (2024). Magnesium alloys as alternative anode materials for rechargeable magnesium-ion batteries: Review on the alloying phase and reaction mechanisms. J. Magnes. Alloy.

[14] Das A., Balakrishnan N., Sreeram P., Fatima M., Joyner J., Thakur V., Pullanchiyodan A., Ahn J.-H., Raghavan P. (2024). Prospects for magnesium ion batteries: A comprehensive materials review. Coord. Chem. Rev..

[15] Malachi I., Olawumi A., Afolabi S., Oladapo B. (2025). Looking Beyond Lithium for Breakthroughs in Magnesium-Ion Batteries as Sustainable Solutions. Sustainability.

[16] Gohar O., Ishfaq H., Iqbal M., Khan M., Basharat S., Kanwal N., Riaz A., Saleem M., Koh J.-H., Hussain I. (2025). Recent advancements in high-performance and durable electrodes materials for magnesium-ion batteries. Coord. Chem. Rev..

[17] Javed M., Shah A., Nisar J., Shahzad S., Haleem A., Shah I. (2024). Nanostructured Design Cathode Materials for Magnesium-Ion Batteries. ACS Omega.

[18] Zhan Y., Zhang W., Lei B., Liu H., Li W. (2020). Recent Development of Mg Ion Solid Electrolyte. Front. Chem..

[19] Javed S., Suo G., Habib L., Lin C., Li J., Hou X., Ye X., Yang Y., Ding S. (2025). Recent progress in zinc-ion battery anodes and cathodes: Materials design, dendrite control, and future perspectives. J. Energy Storage.

[20] Wang M., Meng Y., Li X., Qi J., Li A., Huang S. (2025). Challenges and strategies for zinc anodes in aqueous Zinc-Ion batteries. Chem. Eng. J..

[21] Aniskevich Y., Myung S.-T. (2025). Gains and losses in zinc-ion batteries by proton- and water-assisted reactions. Chem. Soc. Rev..

[22] Hong S., Choi Z., Hwang B., Matic A. (2024). Research Trends and Future Perspectives on Zn-Ion Batteries Using Ga-Based Liquid Metal Coatings on Zn Anodes. ACS Energy Lett..

[23] Wu Y., He Q., Zhou Y., Liu X., Yang M. (2025). Zinc-ion batteries at elevated temperatures: Linking material design to wearable/biocompatible applications. Adv. Compos. Hybrid. Mater..

[24] Uzunoglu G.Y., Yuksel R. (2025). Toward Green and Sustainable Zinc-Ion Batteries: The Potential of Natural Solvent-Based Electrolytes. Small.

[25] Yang Y., Tang Z., Bian S., Gu Y., Ye F., Chen W., Zhu K., Wu Y., Hu L. (2025). Zinc Ion Transport Kinetics in Zinc-based Batteries and Its Regulation Strategy. Adv. Energy Mater..

[26] Deng L., Lin Q., Li Z., Cao J., Sun K., Wei T. (2025). Cathodes for Zinc-Ion Micro-Batteries: Challenges, Strategies, and Perspectives. Batteries.

[27] Zhao J., Chen Y., An Z., Zhang M., Wang W., Guo Q., Li Y., Han S., Zhang L. (2025). Toward the next generation of sustainable aluminum-ion batteries: A review. Green. Chem..

[28] Nandi S., Pumera M. (2025). Materials for aluminum batteries: Progress and challenges. Chem. Eng. J..

[29] Lu C., Wei L., Li J. (2025). Aluminum Ion Batteries: Electrolyte and Anode Innovations and Outlook. Energy Storage Mater..

[30] Wang X., Xi Z., Zhao Q. (2025). Progress on aqueous rechargeable aluminium metal batteries. Ind. Chem. Mater..

[31] Xiao Z., Pang Q. (2025). Transforming Aluminum-Ion Batteries with Recyclable Solid-State Electrolytes. ACS Cent. Sci..

[32] Liu W., Li L., Yue S., Jia S., Wang C., Zhang D. (2024). Electrolytes for Aluminum-Ion Batteries: Progress and Outlook. Chem.A Eur. J..

[33] Melzack N., Wills R. (2022). A Review of Energy Storage Mechanisms in Aqueous Aluminium Technology. Front. Chem. Eng.

[34] Barman M., Pal M., Biswas R., Dutta A. (2025). A comprehensive review of metal-air batteries: Mechanistic aspects, advantages and challenges. Catal. Today.

[35] Li X.-Y., Zhao M., Song Y.-W., Bi C.-X., Li Z., Chen Z.-X., Zhang X.-Q., Li B.-Q., Huang J.-Q. (2025). Polysulfide chemistry in metal–sulfur batteries. Chem. Soc. Rev..

[36] Elia G., Abdelhamid M., Ming J., Jankowski P. (2021). 8—Application of nanotechnology in multivalent ion-based batteries. Front. Nanosci..

[37] da Silva S.B.G., Barros M.V., Radicchi J.Â.Z., Puglieri F.N., Piekarski C.M. (2024). Opportunities and challenges to increase circularity in the product’s use phase. Sustain. Futur..

[38] Picatoste A., Justel D., Mendoza J. (2025). Circular design criteria and indicators for the sustainable life cycle management of electric vehicle batteries. Sustain. Prod. Consum..

[39] Jafarizadeh H., Yamini E., Zolfaghari S.M., Esmaeilion F., Assad M., Soltani M. (2024). Navigating challenges in large-scale renewable energy storage: Barriers, solutions, and innovations. Energy Rep..

[40] Zhu P., Li J., Wang Y., Jin Y. (2025). Multi-Enhanced High-Entropy NASICON Cathodes for High Voltage and Stability in Sodium-Ion Batteries. ACS Appl. Mater. Interfaces.

[41] Mohsin F., Hossain N., Alvy T., Sharmin T., Haque M., Mashfy M.M., Mousa M., Nasim M. (2025). Exploring the limitations and unlocking the potential of sodium-ion battery cathodes. Mater. Today Energy.

[42] Li G., Lou X., Peng C., Liu C., Chen W. (2022). Interface chemistry for sodium metal anodes/batteries: A review. Chem. Synth..

[43] Banerjee S., Choudhary R., Ansari S. (2024). Na3V2(PO4)3 derived cathode materials for sodium-ion batteries (SIBs): A review. Futur. Batter..

[44] Lee S., Kim Y., Park J.-H., Susanto D., Kim J.-Y., Han J., Jun S., Chung K. (2024). Mechanical Activation of Graphite for Na-Ion Battery Anodes: Unexpected Reversible Reaction on Solid Electrolyte Interphase via X-Ray Analysis. Adv. Sci..

[45] Jia Q., Li Z., Ruan H., Luo D., Wang J., Ding Z., Chen L. (2024). A Review of Carbon Anode Materials for Sodium-Ion Batteries: Key Materials, Sodium-Storage Mechanisms, Applications, and Large-Scale Design Principles. Molecules.

[46] Wu J., Liu J., Cui J., Yao S., Ihsan-Ul-Haq M., Mubarak N., Quattrocchi E., Ciucci F., Kim J.-K. (2020). Dual-phase MoS2 as a high-performance sodium-ion battery anode. J. Mater. Chem. A.

[47] Dai H., Tang M., Huang J., Wang Z. (2021). A Series of Molecule-Intercalated MoS2 as Anode Materials for Sodium Ion Batteries. ACS Appl. Mater. Interfaces.

[48] Yang Y., Zheng F., Wang L., Liu Y. (2024). 3D MoS2/graphene oxide integrated composite as anode for high-performance sodium-ion batteries. Sci. Rep..

[49] Zhang Y., Chen J., Kang T., Yang W., Zou H., Chen S. (2024). MoS2 spheres covered with a few layers of MXene as a high-performance anode for sodium-ion batteries. Dalt. Trans..

[50] Ahangari M., Zhou M., Luo H. (2025). Review of Layered Transition Metal Oxide Materials for Cathodes in Sodium-Ion Batteries. Micromachines.

[51] Liu Y., Ullah M., Gao X., Liu P., Li Y., Wang W. (2025). Hierarchical fragmented Na_3_V_2_(PO_4_)_3_@reduced graphene composites with enhanced sodium-ion storage performance. J. Power Sources.

[52] Huang J.-Q., Du R., Zhang H., Liu Y., Chen J., Liu Y.-J., Li L., Peng J., Qiao Y., Chou S.-L. (2023). Low-cost Prussian blue analogues for sodium-ion batteries and other metal-ion batteries. Chem. Commun..

[53] Li H.-W., Wang J., Yu J., Li J.-Y., Zhu Y.-F., Dong H., Zhang Z., Jiang Y., Dou S.-X., Xiao Y. (2025). A universal strategy for bridging Prussian blue analogues and sodium layered oxide cathodes: Direct fast conversion, dynamic structural evolution, and sodium storage mechanisms. Chem. Sci..

[54] Monti D., Jónsson E., Boschin A., Palacín M., Ponrouch A., Johansson P. (2020). Towards standard electrolytes for sodium-ion batteries: Physical properties, ion solvation and ion-pairing in alkyl carbonate solvents. Phys. Chem. Chem. Phys..

[55] Lee D., Kakarla A.K., Sun S., Kim P.J., Choi J. (2025). Inorganic Solid-State Electrolytes for Solid-State Sodium Batteries: Electrolyte Design and Interfacial Challenges. ChemElectroChem.

[56] Li Y., Wang Q., Zhao X., He B., Xiao Y., Guo J., Yang L., Liao R. (2025). High ionic conductive sodium β-alumina (SBA) and SBA-NaPF6 composite solid electrolytes prepared by cold sintering process. Ceram. Int..

[57] Guan S., Lu J., Li Y., Xie D., Zhuang C., Zhang W. (2025). Influence of calcium-doped on the conductivity of NASICON-type Na3Zr2Si2PO4 solid electrolyte. Ceram. Int..

[58] Fujita Y., Yamanaka R., Suehiro D., Imai K., Koga K., Asakura T., Motohashi K., Sakuda A., Hayashi A. (2025). Amorphous Sulfide Solid Electrolytes Based on Na3PS4–NaxMOy (M = P and S) for All-Solid-State Sodium Batteries. ACS Appl. Energy Mater..

[59] Huang Z., Yoshida S., Akamatsu H., Hayashi K., Ohno S. (2024). NaMCl6 (M = Nb and Ta): A New Class of Sodium-Conducting Halide-Based Solid Electrolytes. ACS Mater. Lett..

[60] Gao Y., Yu Q., Yang H., Zhang J., Wang W. (2024). The Enormous Potential of Sodium/Potassium-Ion Batteries as the Mainstream Energy Storage Technology for Large-Scale Commercial Applications. Adv. Mater..

[61] Waseem M., Lakshmi G., Ahmad M., Suhaib M. (2025). Energy storage technology and its impact in electric vehicle: Current progress and future outlook. Next Energy.

[62] Enasel E., Dumitrascu G. (2025). Storage solutions for renewable energy: A review. Energy Nexus.

[63] Yao A., Benson S., Chueh W. (2025). Critically assessing sodium-ion technology roadmaps and scenarios for techno-economic competitiveness against lithium-ion batteries. Nat. Energy.

[64] Wickerts S., Arvidsson R., Nordelöf A., Svanström M., Johansson P. (2024). Prospective life cycle assessment of sodium-ion batteries made from abundant elements. J. Ind. Ecol..

[65] Batuecas E., Martínez-Cisneros C., Serrano D., Várez A. (2024). Life cycle assessment of lab-scale solid sodium-ion batteries: A sustainable alternative to liquid lithium-ion batteries. J. Energy Storage.

[66] Zhang S., Steubing B., Potter H.K., Hansson P.-A., Nordberg Å. (2024). Future climate impacts of sodium-ion batteries. Resour. Conserv. Recycl..

[67] Guo W., Feng T., Li W., Hua L., Meng Z., Li K. (2023). Comparative life cycle assessment of sodium-ion and lithium iron phosphate batteries in the context of carbon neutrality. J. Energy Storage.

[68] Mozaffarpour F., Hassanzadeh N., Vahidi E. (2022). Comparative life cycle assessment of synthesis routes for cathode materials in sodium-ion batteries. Clean. Technol. Environ. Policy.

[69] Taherkhani E., Sabour M., Faraji G. (2024). Sustainable magnesium recycling: Insights into grain refinement through plastic deformation-assisted solid-state recycling (SSR). J. Magnes. Alloy.

[70] Mousavian S.M.H., Bautin V. (2025). Rechargeable magnesium batteries: Overcoming challenges for high-efficiency energy applications. J. Energy Storage.

[71] Luo X., Shen A., Liu B., Wu J., Fan M., Yang N., Zhang G., Chen X., Dai Q. (2025). The interfacial reactions of Mg battery anodes. J. Magnes. Alloy.

[72] Yang J., Li J., Gong W., Geng F. (2021). Genuine divalent magnesium-ion storage and fast diffusion kinetics in metal oxides at room temperature. Proc. Natl. Acad. Sci. USA.

[73] Gao L., Sun C., Li X., Li J., Bian X. (2025). Dynamic regulation of lithium ions eliminating the lithium dendrite formation. Chem. Eng. J..

[74] Prasad S., Prasad S., Verma K., Mishra R., Kumar V., Singh S. (2022). The role and significance of Magnesium in modern day research-A review. J. Magnes. Alloy.

[75] Asif M., Kilian S., Rashad M. (2021). Uncovering electrochemistries of rechargeable magnesium-ion batteries at low and high temperatures. Energy Storage Mater..

[76] Kotobuki M., Yan B., Lu L. (2023). Recent progress on cathode materials for rechargeable magnesium batteries. Energy Storage Mater..

[77] Ramasubramanian B., Reddy M., Zaghib K., Armand M., Ramakrishna S. (2021). Growth Mechanism of Micro/Nano Metal Dendrites and Cumulative Strategies for Countering Its Impacts in Metal Ion Batteries: A Review. Nanomaterials.

[78] Wang L., Li P.C., Family R., Detsi E. (2023). Magnesium dendrite growth during electrodeposition in conditioned Mg(TFSI)_2_/AlCl_3_/MgCl_2_/DME electrolyte. J. Nanoparticle Res..

[79] Attari V., Banerjee S., Arroyave R. (2024). On the kinetics of electrodeposition in a magnesium metal anode. Acta Mater..

[80] Yan B., Karuppiah C., Walle K., Abdelaal M., Kotobuki M., Lu L. (2024). Review on dendrite formation of Mg metal anode and its prevention. Nano Energy.

[81] Davidson R., Verma A., Santos D., Hao F., Fincher C., Xiang S., Van Buskirk J., Xie K., Pharr M., Mukherjee P. (2019). Formation of Magnesium Dendrites during Electrodeposition. ACS Energy Lett..

[82] Chen X., Wei S., Tong F., Taylor M.P., Cao P. (2021). Electrochemical performance of Mg-Sn alloy anodes for magnesium rechargeable battery. Electrochim. Acta.

[83] Saritha D. (2023). A mini review on alloy-based anode materials for Mg-ion batteries. Mater. Today Proc..

[84] Wu C., Xue L., Xu R., Fan J., Chen T., Tang W., Cui L., Wang A., Dou S., Peng C. (2024). Toward high-energy magnesium battery anode: Recent progress and future perspectives. Mater. Today Energy.

[85] Cen Y., Dong J., Zhu T., Cai X., Wang X., Hu B., Xu C., Yu D., Liu Y., Chen C. (2021). Bi nanorods anchored in N-doped carbon shell as anode for high-performance magnesium ion batteries. Electrochim. Acta.

[86] Pan S., Cheng M., Ma C., Jing H., Shen T., Hu J., Liu Q., Wei T., Wang R., Li W. (2025). Bimetallic Bi–Sn nanoparticles in-situ anchored in carbon nanofiber as flexible self-supporting anode toward advanced magnesium ion batteries. Chem. Eng. J..

[87] Liu M., Lv G., Liu T., Liu H., Kong L., Tian L., Rao W., Li Y., Liao L., Guo J. (2023). Chevrel phase: A review of its crystal structure and electrochemical properties. Prog. Nat. Sci. Mater. Int..

[88] Mizanuzzaman M., Chowdhury M.A., Khandaker T., Islam M., Abdullah M. (2025). Advancing Cathode Materials for Rechargeable Magnesium-Ion Batteries: Sustainable Structures, Ecofriendly Properties, and Enhanced Electrochemical Performance. Energy Technol..

[89] Tolstopyatova E.G., Salnikova Y.D., Holze R., Kondratiev V.V. (2024). Progress and Challenges of Vanadium Oxide Cathodes for Rechargeable Magnesium Batteries. Molecules.

[90] Pryke J., Kennard R., Cussen S. (2022). Cathodes for Mg batteries: A condensed review. Energy Rep..

[91] Man Y., Jaumaux P., Xu Y., Fei Y., Mo X., Wang G., Zhou X. (2023). Research development on electrolytes for magnesium-ion batteries. Sci. Bull..

[92] Pang Y., Zhu Y., Fang F., Sun D., Zheng S. (2023). Advances in solid Mg-ion electrolytes for solid-state Mg batteries. J. Mater. Sci. Technol..

[93] Chinnadurai D., Lieu W., Kumar S., Yang G., Li Y., Seh Z. (2023). A Passivation-Free Solid Electrolyte Interface Regulated by Magnesium Bromide Additive for Highly Reversible Magnesium Batteries. Nano Lett..

[94] Jeon A.-R., Jeon S., Lim G., Jang J., No W.J., Oh S.H., Hong J., Yu S.-H., Lee M. (2023). Reversible Magnesium Metal Cycling in Additive-Free Simple Salt Electrolytes Enabled by Spontaneous Chemical Activation. ACS Nano.

[95] Li Y., Feng X., Yang G., Lieu W.Y., Fu L., Zhang C., Xing Z., Ng M.-F., Zhang Q., Liu W. (2024). Toward waterproof magnesium metal anodes by uncovering water-induced passivation and drawing water-tolerant interphases. Nat. Commun..

[96] Zhang H., Qiao L., Armand M. (2022). Organic Electrolyte Design for Rechargeable Batteries: From Lithium to Magnesium. Angew. Chem. Int. Ed..

[97] Sun Q., Luo S., Huang R., Yan S., Lin X. (2024). Recent progress of magnesium electrolytes for rechargeable magnesium batteries. Coord. Chem. Rev..

[98] Chen F., Meng Q., Wang H., Yu J., Li R., Yi Y., Hua Y., Lin H., Jiang P., Chan K. (2025). Glycol-glyme co-solvent electrolytes enable high-capacity and ultrastable VO2 cathodes in magnesium ion batteries. Nano Energy.

[99] Zhang D., Duan S., Liu X., Yang Y., Zhang Y., Ren W., Zhang S., Cheng M., Yang W., Wang J. (2023). Deeping insight of Mg(CF3SO3)2 and comprehensive modified electrolyte with ionic liquid enabling high-performance magnesium batteries. Nano Energy.

[100] Shah R., Mittal V., Matsil E., Rosenkranz A. (2021). Magnesium-ion batteries for electric vehicles: Current trends and future perspectives. Adv. Mech. Eng..

[101] Blázquez J., Maça R., Leonet O., Azaceta E., Mukherjee A., Zhao-Karger Z., Li Z., Kovalevsky A., Fernández-Barquín A., Mainar A. (2023). A practical perspective on the potential of rechargeable Mg batteries. Energy Environ. Sci..

[102] Pathak A., Cha E., Choi W. (2024). Towards the commercialization of Li-S battery: From lab to industry. Energy Storage Mater..

[103] Cao H., Zhu Y., Jiang T., Leng Z., Yu H., Ma X., Cao C., Zou M. (2025). Fundamental Understanding and Material Challenges in Rechargeable Magnesium-Sulfur Battery: Current Advances and Perspective. Small.

[104] Pinto-Bautista S., Baumann M., Weil M. (2024). Prospective life cycle assessment of an electric vehicle equipped with a model magnesium battery. Energy. Sustain. Soc..

[105] Montenegro C.T., Peters J., Baumann M., Zhao-Karger Z., Wolter C., Weil M. (2021). Environmental assessment of a new generation battery: The magnesium-sulfur system. J. Energy Storage.

[106] Alawi M., Gamal H., Rashad M., Alziyadi M., Shalaby M. (2025). inc-ion batteries: Drawbacks, opportunities, and optimization performance for sustainable energy storage. J. Alloys Compd..

[107] Li B., Ma Y., Ma J., Chen L., Zhao Y., Tang M.-C. (2024). Challenges and opportunities facing zinc anodes for aqueous zinc-ion battery. Energy Mater. Devices.

[108] Tao F., Liu Y., Ren X., Wang J., Zhou Y., Miao Y., Ren F., Wei S., Ma J. (2022). Different surface modification methods and coating materials of zinc metal anode. J. Energy Chem..

[109] An Y., Xu B., Tian Y., Shen H., Man Q., Liu X., Yang Y., Li M. (2023). Reversible Zn electrodeposition enabled by in-terfacial chemistry manipulation for high-energy anode-free Zn batteries. Mater. Today.

[110] Qi B., Huang M., Song M., Zhou W., Tan H. (2025). Alloying Design Strategies for High-Performance Zn Anodes in Aqueous Zinc-Ion Batteries. Materials.

[111] Fan C., Meng W., Ye J. (2024). Towards advanced zinc anodes by interfacial modification strategies for efficient aqueous zinc metal batteries. J. Energy Chem..

[112] Wang D., Li Q., Zhao Y., Hong H., Li H., Huang Z., Liang G., Yang Q., Zhi C. (2022). Insight on Organic Molecules in Aqueous Zn-Ion Batteries with an Emphasis on the Zn Anode Regulation. Adv. Energy Mater..

[113] Zheng J., Archer L. (2025). Controlling electrochemical growth of metallic zinc electrodes: Toward affordable rechargeable energy storage systems. Sci. Adv..

[114] Hao J., Li X., Zeng X., Li D., Mao J., Guo Z. (2020). Deeply understanding the Zn anode behaviour and corresponding improvement strategies in different aqueous Zn-based batteries. Energy Environ. Sci..

[115] Yuan L., Hao J., Kao C.-C., Wu C., Liu H.-K., Dou S.-X., Qiao S.-Z. (2021). Regulation methods for the Zn/electrolyte interphase and the effectiveness evaluation in aqueous Zn-ion batteries. Energy Environ. Sci..

[116] Zhang Y., Zheng X., Wang N., Lai W.-H., Liu Y., Chou S.-L., Liu H.-K., Dou S.-X., Wang Y.-X. (2022). Anode optimization strategies for aqueous zinc-ion batteries. Chem. Sci..

[117] Zhao R., Yang Y., Liu G., Zhu R., Huang J., Chen Z., Gao Z., Chen X., Qie L. (2021). Redirected Zn Electrodeposition by an Anti-Corrosion Elastic Constraint for Highly Reversible Zn Anodes. Adv. Funct. Mater..

[118] He W., Wang S., Shao Y., Kong Z., Tu H., Wu Y., Hao X. (2021). Water Invoking Interface Corrosion: An Energy Density Booster for Ni//Zn Battery. Adv. Energy Mater..

[119] Wang Y., Wang Z., Yang F., Liu S., Zhang S., Mao J., Guo Z. (2022). Electrolyte Engineering Enables High Performance Zinc-Ion Batteries. Small.

[120] Wang L., Yan S., Quilty C., Kuang J., Dunkin M., Ehrlich S., Ma L., Takeuchi K., Takeuchi E., Marschilok A. (2021). Achieving Stable Molybdenum Oxide Cathodes for Aqueous Zinc-Ion Batteries in Water-in-Salt Electrolyte. Adv. Mater. Interfaces.

[121] Bayaguud A., Luo X., Fu Y., Zhu C. (2020). Cationic Surfactant-Type Electrolyte Additive Enables Three-Dimensional Dendrite-Free Zinc Anode for Stable Zinc-Ion Batteries. ACS Energy Lett..

[122] Hao J., Long J., Li B., Li X., Zhang S., Yang F., Zeng X., Yang Z., Pang W.K., Guo Z. (2019). Toward High-Performance Hybrid Zn-Based Batteries via Deeply Understanding Their Mechanism and Using Electrolyte Additive. Adv. Funct. Mater..

[123] Sun P., Ma L., Zhou W., Qiu M., Wang Z., Chao D., Mai W. (2021). Simultaneous Regulation on Solvation Shell and Electrode Interface for Dendrite-Free Zn Ion Batteries Achieved by a Low-Cost Glucose Additive. Angew. Chem..

[124] Hao J., Li X., Zhang S., Yang F., Zeng X., Zhang S., Bo G., Wang C., Guo Z. (2020). Designing Dendrite-Free Zinc Anodes for Advanced Aqueous Zinc Batteries. Adv. Funct. Mater..

[125] Li H., Han C., Huang Y., Huang Y., Zhu M., Pei Z., Xue Q., Wang Z., Liu Z., Tang Z. (2018). An extremely safe and wearable solid-state zinc ion battery based on a hierarchical structured polymer electrolyte. Energy Environ. Sci..

[126] Deng W., Zhou Z., Li Y., Zhang M., Yuan X., Hu J., Li Z., Li C., Li R. (2020). High-Capacity Layered Magnesium Vanadate with Concentrated Gel Electrolyte toward High-Performance and Wide-Temperature Zinc-Ion Battery. ACS Nano.

[127] Zhang S., Yu N., Zeng S., Zhou S., Chen M., Di J., Li Q. (2018). An adaptive and stable bio-electrolyte for rechargeable Zn-ion batteries. J. Mater. Chem. A.

[128] Xie S., Li Y., Li X., Zhou Y., Dang Z., Rong J., Dong L. (2021). Stable Zinc Anodes Enabled by Zincophilic Cu Nanowire Networks. Nano-Micro Lett..

[129] Gourley S., Brown R., Adams B., Higgins D. (2023). Zinc-ion batteries for stationary energy storage. Joule.

[130] Ugalde C., Gonzalez C., Vera M., García J., Velázquez W. Performance analysis of a novel Zinc-air battery powering an IoT node. Proceedings of the 2022 IEEE International Autumn Meeting on Power, Electronics and Computing (ROPEC).

[131] Mageto T., Bhoyate S., Mensah-Darkwa K., Kumar A., Gupta R. (2023). Development of high-performance zinc-ion batteries: Issues, mitigation strategies, and perspectives. J. Energy Storage.

[132] Borchers N., Clark S., Horstmann B., Jayasayee K., Juel M., Stevens P. (2021). Innovative zinc-based batteries. J. Power Sources.

[133] Santos F., Urbina A., Abad J., López R., Toledo C., Romero A.F. (2020). Environmental and economical assessment for a sustainable Zn/air battery. Chemosphere.

[134] Iturrondobeitia M., Akizu-Gardoki O., Amondarain O., Minguez R., Lizundia E. (2022). Environmental Impacts of Aqueous Zinc Ion Batteries Based on Life Cycle Assessment. Adv. Sustain. Syst..

[135] Grignon E., Battaglia A., Schon T., Seferos D. (2022). Aqueous zinc batteries: Design principles toward organic cathodes for grid applications. IScience.

[136] Elia G.A., Kravchyk K., Kovalenko M., Chacón J., Holland A., Wills R. (2021). An overview and prospective on Al and Al-ion battery technologies. J. Power Sources.

[137] Guo K., Wang W., Song W.-L., Li S., Du X., Jiao S. (2025). A Recyclable Inert Inorganic Framework Assisted Solid-State Electrolyte for Long-Life Aluminum Ion Batteries. ACS Cent. Sci..

[138] Alfaruqi M., Islam S., Lee J., Jo J., Mathew V., Kim J. (2019). First principles calculations study of α-MnO2 as a potential cathode for Al-ion battery application. J. Mater. Chem. A.

[139] Pan W., Zhang Y., Leong K.W., Zhang Y., Mao J., Wang Y., Zhao X., Luo S., Leung D. (2024). Unlocking the Potential of 2D MoS2 Cathodes for High-Performance Aqueous Al-Ion Batteries: Deciphering the Intercalation Mechanisms. Small Methods.

[140] Hu Y., Li H., Zhao X., Zhang W., Li Z. (2024). Organometallic structure-based βCD@FeSe2/CoSe2 heterostructures cathodes for high-performance aluminum-ion battery. Scr. Mater..

[141] Yang Y., Zhou J., Wang L., Jiao Z., Xiao M., Huang Q., Liu M., Shao Q., Sun X., Zhang J. (2022). Prussian blue and its analogues as cathode materials for Na-, K-, Mg-, Ca-, Zn- and Al-ion batteries. Nano Energy.

[142] Grindal A., Azimi G. (2024). Advancing aluminum-ion batteries: Unraveling the charge storage mechanisms of cobalt sulfide cathodes. Sci. Rep..

[143] Li L., Jia S., Shi Y., Wang C., Qiu H., Ji Y., Cao M., Zhang D. (2024). Recent progress in aluminum anodes for high-performance rechargeable aqueous Al-ion batteries. Inorg. Chem. Front..

[144] Lu C., Wang Z., Gao J., Li J., Wei L. (2024). Synergistic Effect of Anodic Hydrophilic and Hydrophobic Interfaces for Long Cycle Life Aqueous Aluminum–Zinc Hybrid Ion Batteries. Adv. Energy Mater..

[145] Korzekwa J. (2023). Modification of the structure and properties of oxide layers on aluminium alloys: A review. Rev. Adv. Mater. Sci..

[146] Gao T., Li X., Wang X., Hu J., Han F., Fan X., Suo L., Pearse A.J., Lee S.B., Rubloff G.W. (2016). A Rechargeable Al/S Battery with an Ionic-Liquid Electrolyte. Angew. Chem. Int. Ed..

[147] Craig B., Schoetz T., Cruden A., Ponce de Leon C. (2020). Review of current progress in non-aqueous aluminium batteries. Renew. Sustain. Energy Rev..

[148] Rehman W.U., Huang H., Yousaf M.Z., Aslam F., Wang X., Ghani A. (2023). Porous Carbon with Alumina Coating Nanolayer Derived from Biomass and the Enhanced Electrochemical Performance as Stable Anode Materials. Molecules.

[149] Zheng X., Xie Y., Tian F., Lei D., Wang C. (2024). 3D porous reduced graphene cathode and non-corrosive electrolyte for long-life rechargeable aluminum batteries. Energy Mater. Devices.

[150] Muñoz-Torrero D., Palma J., Marcilla R., Ventosa E. (2019). A critical perspective on rechargeable Al-ion battery technology. Dalt. Trans..

[151] Liu Z., Feng F., Feng W., Wang G., Qi B., Gong M., Zhang F., Pang H. (2025). Eutectic electrolytes: A new platform for high-safety batteries. Energy Environ. Sci..

[152] Wang H., Gao Y., Li Y., Yang H., Liu W., Long B., Wu F., Bai Y., Wu C. (2025). Metal Aluminum-Free Configuration Toward High-Performance Aqueous Aluminum Ion Battery. Energy Mater. Adv..

[153] Ng K.L., Amrithraj B., Azimi G. (2022). Nonaqueous rechargeable aluminum batteries. Joule.

[154] Meng P., Yang Z., Zhang J., Jiang M., Wang Y., Zhang X., Luo J., Fu C. (2023). Electrolyte design for rechargeable aluminum-ion batteries: Recent advances and challenges. Energy Storage Mater..

[155] Raj M.R., Zaghib K., Lee G. (2025). Advanced aqueous electrolytes for aluminum-ion batteries: Challenges and opportunities. Energy Storage Mater..

[156] Gupta S., Vishwakarma J., Srivastava A., Dhand C., Dwivedi N. (2024). Aluminum batteries: Opportunities and challenges. Energy Storage Mater..

[157] Delgado M., Usai L., Ellingsen L., Pan Q., Strømman A. (2019). Comparative Life Cycle Assessment of a Novel Al-Ion and a Li-Ion Battery for Stationary Applications. Materials.

[158] Melzack N., Wills R., Cruden A. (2021). Cleaner Energy Storage: Cradle-to-Gate Life Cycle Assessment of Aluminum-Ion Batteries with an Aqueous Electrolyte. Front. Energy Res..

[159] Melzack N. (2022). Advancing battery design based on environmental impacts using an aqueous Al-ion cell as a case study. Sci. Rep..

[160] Mączka M., Guzik M., Mosiałek M., Wojnarowska M., Pasierb P., Nitkiewicz T. (2024). Life cycle assessment of experimental Al-ion batteries for energy storage applications. Sci. Total Environ..

[161] Qahtan T., Alade I., Rahaman M., Alansi A., Saleh T. (2025). Trends in metal-air battery research: Clusters, and future directions. J. Alloys Compd..

[162] Cao W., Zhang J., Li H. (2020). Batteries with high theoretical energy densities. Energy Storage Mater..

[163] Liu W., Li Y., Yang H., Long B., Li Y., Bai Y., Wu C., Wu F. (2023). Pursuing high voltage and long lifespan for low-cost Al-based rechargeable batteries: Dual-ion design and prospects. Energy Storage Mater..

[164] Jia H., Wang Z., Tawiah B., Wang Y., Chan C.-Y., Fei B., Pan F. (2020). Recent advances in zinc anodes for high-performance aqueous Zn-ion batteries. Nano Energy.

[165] Tarroja B., Ogunseitan O., Kendall A., Passerini S., Barelli L., Baumann M., Peters J., Weil M. (2024). Life Cycle Assessment of Emerging Battery Systems. Emerging Battery Technologies to Boost the Clean Energy Transition: Cost, Sustainability, and Performance Analysis.

[166] Hosseini S., Soltani S., Li Y.-Y. (2021). Current status and technical challenges of electrolytes in zinc–air batteries: An in-depth review. Chem. Eng. J..

[167] Rani B., Yadav J., Saini P., Pandey A., Dixit A. (2024). Aluminum–air batteries: Current advances and promises with future directions. RSC Adv..

[168] Wang Y., Sun Y., Ren W., Zhang D., Yang Y., Yang J., Wang J., Zeng X., NuLi Y. (2022). Challenges and prospects of Mg-air batteries: A review. Energy Mater..

[169] Chawla N., Safa M. (2019). Sodium Batteries: A Review on Sodium-Sulfur and Sodium-Air Batteries. Electronics.

[170] Barbosa J., Pinto R., Serra J., Gonçalves R., Lizundia E., Costa C., Lanceros-Mendez S. (2025). Batteries and sustainability: The relevance of life cycle assessment. Sustain. Energy Technol. Assess..

[171] Zackrisson M., Fransson K., Hildenbrand J., Lampic G., O’Dwyer C. (2016). Life cycle assessment of lithium-air battery cells. J. Clean. Prod..

[172] Iturrondobeitia M., Akizu-Gardoki O., Minguez R., Lizundia E. (2021). Environmental Impact Analysis of Aprotic Li–O2 Batteries Based on Life Cycle Assessment. ACS Sustain. Chem. Eng..

[173] Yang S., Knickle H. (2002). Design and analysis of aluminum/air battery system for electric vehicles. J. Power Sources.

[174] Ji W., Du D., Liang J., Li G., Feng G., Yin Z., Zhou J., Zhao J., Shen Y., Huang H. (2023). Aqueous Zn−organic batteries: Electrochemistry and design strategies. Batter. Energy.

[175] Tian Y., Pei Z., Luan D., Lou W. (2025). Anchoring Sn Nanoparticles in Necklace-Like B,N,F-Doped Carbon Fibers Enables Anode-Less 5V-Class Li-Metal Batteries. Angew. Chem. Int. Ed..

[176] Lavisse T., Panariello R., Perdu F., Zwolinski P. (2023). Integrating an ageing model within Life Cycle Assessment to evaluate the environmental impacts of electric batteries. Procedia CIRP.

[177] Erakca M., Bautista S.P., Moghaddas S., Baumann M., Bauer W., Leuthner L., Weil M. (2023). Closing gaps in LCA of lithium-ion batteries: LCA of lab-scale cell production with new primary data. J. Clean. Prod..

[178] Rezaei M., Nekahi A., Kumar A., Nizami A., Li X., Deng S., Nanda J., Zaghib K. (2025). A review of lithium-ion battery recycling for enabling a circular economy. J. Power Sources.

[179] Abdalla A., Abdullah M., Dawood M., Wei B., Subramanian Y., Azad A.T., Nourin S., Afroze S., Taweekun J., Azad A. (2023). Innovative lithium-ion battery recycling: Sustainable process for recovery of critical materials from lithium-ion batteries. J. Energy Storage.

[180] Šimaitis J., Allen S., Vagg C. (2023). Are future recycling benefits misleading? Prospective life cycle assessment of lithium-ion batteries. J. Ind. Ecol..

[181] Llamas-Orozco J. Dynamic Life Cycle Assessment of Lithium-Ion Batteries for Electric Vehicles. Ph.D. Thesis.

[182] Machala M., Chen X., Bunke S., Forbes G., Yegizbay A., de Chalendar J., Azevedo I., Benson S., Tarpeh W. (2025). Life cycle comparison of industrial-scale lithium-ion battery recycling and mining supply chains. Nat. Commun..

[183] Scrucca F., Presciutti A., Baldinelli G., Barberio G., Postrioti L., Karaca C. (2025). Life cycle assessment of Li-ion batteries for electric vehicles: A review focused on the production phase impact. J. Power Sources.

[184] Dolganova I., Rödl A., Bach V., Kaltschmitt M., Finkbeiner M. (2020). A Review of Life Cycle Assessment Studies of Electric Vehicles with a Focus on Resource Use. Resources.

[185] Wei G., Liu Y., Jiao B., Chang N., Wu M., Liu G., Lin X., Weng X., Chen J., Zhang L. (2023). Direct recycling of spent Li-ion batteries: Challenges and opportunities toward practical applications. IScience.

[186] Koroma M., Costa D., Philippot M., Cardellini G., Hosen M.S., Coosemans T., Messagie M. (2022). Life cycle assessment of battery electric vehicles: Implications of future electricity mix and different battery end-of-life management. Sci. Total Environ..

[187] Verma S., Dwivedi G., Verma P. (2022). Life cycle assessment of electric vehicles in comparison to combustion engine vehicles: A review. Mater. Today Proc..

[188] Song H., Chen H., Wang Y., Sun X.-E. (2024). An Overview About Second-Life Battery Utilization for Energy Storage: Key Challenges and Solutions. Energies.

[189] Kampker A., Heimes H., Offermanns C., Vienenkötter J., Frank M., Holz D. (2023). Identification of Challenges for Second-Life Battery Systems—A Literature Review. World Electr. Veh. J..

[190] Das P. (2025). A Perspective on the Challenges and Prospects of Realizing the Second Life of Retired EV Batteries. Batteries.

[191] Yu X., Li W., Gupta V., Gao H., Tran D., Sarwar S., Chen Z. (2022). Current Challenges in Efficient Lithium-Ion Batteries’ Recycling: A Perspective. Glob. Chall..

[192] Roy J., Phuong D., Verma V., Chaudhary R., Carboni M., Meyer D., Cao B., Srinivasan M. (2024). Direct recycling of Li-ion batteries from cell to pack level: Challenges and prospects on technology, scalability, sustainability, and economics. Carbon. Energy.

[193] Lin J., Li W., Chen Z. (2025). Scaling Direct Recycling of Lithium-Ion Batteries toward Industrialization: Challenges and Opportunities. ACS Energy Lett..

[194] Arellano-Sanchez D., Rinne M., Wilson B., Lundström M. (2025). Life cycle assessment of LTO-rich anode waste from lithium-ion battery with a hazardous waste management approach. Resour. Conserv. Recycl..

[195] Peters J.F. (2023). Best practices for life cycle assessment of batteries. Nat. Sustain..

[196] Porzio J., Scown C. (2021). Life-Cycle Assessment Considerations for Batteries and Battery Materials. Adv. Energy Mater..

[197] Haupt J., Kononova N., Cerdas F., Zellmer S., Herrmann C. (2023). Challenges of prospective life cycle assessment of emerging recycling processes: Case study of battery materials recovery. Procedia CIRP.

[198] Song J., Cui G., Han Y., Yao X., Shen X., Wang Y. Review on environmental impacts of various types of power batteries using LCA. Environ. Dev. Sustain..

[199] Pellow M., Ambrose H., Mulvaney D., Betita R., Shaw S. (2020). Research gaps in environmental life cycle assessments of lithium ion batteries for grid-scale stationary energy storage systems: End-of-life options and other issues. Sustain. Mater. Technol..

[200] Liu Y., Liu C., Liu Y., Sun F., Qiao J., Xu T. (2023). Review on degradation mechanism and health state estimation methods of lithium-ion batteries. J. Traffic. Transp. Eng. (Engl. Ed.).

[201] Rehman S., Al-Greer M., Burn A., Short M., Cui X. (2025). High-Volume Battery Recycling: Technical Review of Challenges and Future Directions. Batteries.

[202] (2006). In: Environmental Management–Life Cycle Assessment–Requirements and Guidelines.

[203] Eltohamy H., van Oers L., Lindholm J., Raugei M., Lokesh K., Baars J., Husmann J., Hill N., Istrate R., Jose D. (2024). Review of current practices of life cycle assessment in electric mobility: A first step towards method harmonization. Sustain. Prod. Consum..

[204] Akasapu U., Hehenberger P. (2023). A design process model for battery systems based on existing life cycle assessment results. J. Clean. Prod..

[205] Yan W., Wang X., Liu Y., Zhang X., Jiang Z., Huang L. (2024). A stochastic programming approach for EOL electric vehicle batteries recovery network design under uncertain conditions. Sci. Rep..

[206] Nanaki E. (2021). Chapter 5-Climate change mitigation and electric vehicles. Electric Vehicles for Smart Cities:: Trends, Challenges, and Opportunities.

[207] Mariev O., Islam M.M. (2025). The impact of financial stress, governance, and geopolitics on Europe’s energy transition mineral trade. Energy Econ..

[208] Bednarski L., Roscoe S., Blome C., Schleper M. (2025). Geopolitical disruptions in global supply chains: A state-of-the-art literature review. Prod. Plan. Control.

[209] Meng Z., Sun H., Daigo I., Guan Y., Shan Y. (2025). Technological risks disrupting trade stability in the global lithium supply chain network. IScience.

